# Bitter gourd peptides (BG) alleviate lupus progression in mice through regulation of miR-146a/BRD4 axis in macrophages

**DOI:** 10.3389/fimmu.2026.1666212

**Published:** 2026-02-13

**Authors:** Yu Wu, Wenyan Han, Xian Li, Xiulan Su

**Affiliations:** 1 Clinical Medical Research Center of the Affiliated Hospital, Inner Mongolia Medical University, Hohhot, China; 2 Clinical Laboratory, the Second Affiliated Hospital of Inner Mongolia Medical University, Hohhot, China

**Keywords:** bitter gourd (BG), BRD4, macrophages, mir-146a, systemic lupus erythematosus

## Abstract

Bitter gourd peptides (BG) possess anti-inflammatory properties. Macrophages play a pivotal role in systemic lupus erythematosus (SLE). This study aimed to evaluate the therapeutic effect of BG in lupus-prone mice and to investigate its mechanism of action via macrophage modulation. MRL/lpr mice were treated with BG, and disease indicators were assessed. *In vitro*, an LPS-primed, THP-1-derived macrophage model was established and treated with BG. Macrophage polarization and autophagy were analyzed by flow cytometry, Western blot, and immunofluorescence. The role of the miR-146a/BRD4 axis was examined using qPCR, dual-luciferase reporter assay, and gain/loss-of-function approaches. BG treatment alleviated lupus symptoms in mice, including renal pathology, autoantibody production, and inflammation. In tissues, BG promoted a shift in macrophage phenotype from M1- to M2-like and enhanced autophagic activity. *In vitro*, BG inhibited M1-like polarization, promoted an M2-like phenotype, and enhanced autophagic flux. Mechanistically, BG upregulated miR-146a, which targeted and inhibited BRD4. Both miR-146a inhibition and BRD4 overexpression reversed the cellular effects of BG on polarization and autophagy. BG mitigates lupus progression in mice, and its effects are linked to the modulation of macrophage phenotype and autophagic activity, a process associated with the miR-146a/BRD4 axis. These findings highlight a potential therapeutic avenue for SLE.

## 1 Introduction

Systemic lupus erythematosus (SLE) is a systemic autoimmune disease that causes great harm to patients. SLE patients present with immune infiltration and inflammatory damage of several organs (1). SLE is one of the leading causes of death in young women. In a meta-analysis analyzing more than 26,000 female SLE patients, it was found that the all-cause mortality rate was 2.6 times higher than that of the general population (2). The innate immune system leads to the onset of SLE. It initiates an inflammatory cascade, and continues to promote adaptive immune response during disease development. Therefore, targeting the innate immune system is a promising method for SLE treatment (3). As an important part of the innate immune system, macrophages are involved in a variety of physiological processes, including immune tolerance, inflammatory response, and angiogenesis (4). Macrophages have significant phenotypic heterogeneity and are polarized by microbial stimuli, tissue injury and changes in the tissue microenvironment into two phenotypes: classically activated/inflammatory (M1) macrophages and alternatively activated (M2) macrophages. Lipopolysaccharides (LPS) mainly induce M1-type polarization and secretion of pro-inflammatory factors (IL-6, IL-8, TNF-α), while IL-4 induces M2-type polarization and participates in tissue remodeling and immunomodulation, with secretion of anti-inflammatory cytokines, such as IL-10 (5). Autophagy is an important and conserved process to eliminate damaged organelles, protein aggregates and invading pathogens in eukaryotes (6). Autophagy is involved in the evolution and progression of autoimmune diseases. For example, autophagic proteins in rheumatoid arthritis contribute to the pathological citrullination of autoantigens. The increase of type I interferon production in SLE is related to excessive autophagy in plasma cell-like dendritic cells (7). For macrophages, activation of autophagy can promote M2 polarization and inhibit inflammatory response (8). This characteristic of macrophages makes them potential participants in the development of inflammatory and autoimmune diseases. Targeted regulation of macrophage polarization is a promising approach for SLE treatment.

The low toxicity and low side effects of bioactive peptides make them suitable alternatives to toxic chemotherapeutic drugs. As a short amino acid sequence, it possesses multiple physiological functions of the parent protein (9). Currently, the exploration of bioactive peptides is predominantly centered on their anti-tumor properties. These peptides exert their tumor-suppressing effects through a variety of mechanisms, including disrupting cell membranes, impairing mitochondrial function, activating the p53 pathway, and inhibiting angiogenesis. Illustrative examples include peptides isolated from shark cartilage (10), the CS5931 peptide sequence (11), and RALGWSCL, a peptide derived from ginger (12). Furthermore, certain peptides possess a range of beneficial properties including anti-inflammatory, antibacterial, antioxidant, and immunomodulatory effects. Notable examples include the porcine cathelicidin peptide (13) and the PEP1 peptide, which is composed of amino acids (14). Active peptides hold vast potential for applications in both the food and pharmaceutical industries. Aplidine, also known as Romidepsin, a cyclic depeptide derived from marine sponges, has been approved by the U.S. Food and Drug Administration (FDA) for the treatment of cutaneous T-cell lymphoma (15). Despite these advancements, research on the use of peptides for treating autoimmune diseases remains relatively scarce. Bitter gourd biological active extracts have demonstrated promising anti-tumor properties. The mechanism of action for this substance encompasses immune regulation and the mitigation of inflammatory responses (16). We have extracted the bitter gourd peptide (BG) from bitter melon using enzymatic hydrolysis and ultrafiltration techniques, and in our preliminary studies, we have confirmed that it alleviates arthritis in rats by regulating the necroptosis/neutrophil extracellular traps/inflammation axis (17). In light of these attributes, we have conducted pioneering research to investigate its potential effects on SLE for the first time.

microRNAs (miRNAs) are a class of small non-coding RNAs that play a regulatory role in cell activities including growth, differentiation, development, and apoptosis, and can downregulate expression of target genes after transcription. Studies have shown that miR-146a is involved in the regulation of NF-κB in the classical pro-inflammatory pathway. miR-146a inhibits inflammatory responses by targeting TRAF6 and IRAK1 transcripts, which promote NF-κB activity in the TLR signaling pathway (4). Overexpression of miR-146a reduces the production of inflammatory cytokines. In addition, studies have shown that miR-146a can regulate the metabolism of mouse macrophages (18). Bromodomain-containing protein 4 (BRD4) is a transcriptional regulator that plays an important role in autoimmune and inflammatory diseases. Studies have shown that inhibition of BRD4 can inhibit the differentiation of peripheral plasma cells and has the potential to treat systemic lupus erythematosus (SLE). Here, we assessed the efficacy of BG for the treatment of lupus and explored the related molecular mechanisms. We found that BG up-regulated the expression of miR-146a to inhibit the expression of BRD4 and secretion of inflammatory cytokines by M1 macrophages. In addition, BG mediated M2 macrophage polarization and autophagy by regulating the miR-146a/BRD4 axis, thereby alleviating lupus in mice.

## 2 Materials and methods

### 2.1 Bitter gourd peptides preparation

BG is obtained from the whole plant of bitter gourd through enzymatic hydrolysis and ultrafiltration techniques, provided by the Inner Mongolia Bioactive Peptide Engineering Laboratory. Currently, the BG has been granted a national invention patent.

### 2.2 Animals

A total of twenty 8-week-old female MRL/lpr lupus mice and ten 6–8-week-old female MRL/MpJ mice were purchased from Changzhou Cavens Experimental Animal Co., Ltd. Upon arrival, all mice were housed under specific pathogen-free (SPF) conditions with a controlled room temperature of 20°C, relative humidity of 40%–60%, a 12-h light/dark cycle, and free access to food and water. The MRL/lpr mice were randomly divided into two experimental groups (n=10 per group): (1) the Lupus Model group, receiving daily intragastric administration of normal saline; (2) the BG Treatment group, receiving daily intragastric administration of BG (BG dosage is 0.06g/kg). The MRL/MpJ mice (n=10) served as the Normal Control group and received normal saline following the same schedule. The intervention lasted for 3 weeks. Characteristics and survival of the experimental mouse groups are shows in **Table 1**.

**Table 1:** Characteristics and survival of the experimental mouse groups.

Group	Strain	Initial n	Final n	Survival rate	Key observed complications (Incidence)	Treatment (duration)
Normal Control	MRL/MpJ	10	10	100%	None	Normal saline, i.g., 3 weeks
Lupus Model	MRL/lpr	10	10	100%	Cutaneous lesions (mild to moderate), visible lymphadenopathy	Normal saline, i.g., 3 weeks
BG Treatment	MRL/lpr	10	10	100%	Cutaneous lesions (mild), visible lymphadenopathy	BG (0.06 g/kg), i.g., 3 weeks

#### 2.2.1 Animal monitoring and sample collection

Mice were monitored daily for general health, activity, body weight, and the development of visible lupus-associated complications, such as cutaneous lesions (alopecia, erythema) and signs of distress. Throughout the 3-week study period, all mice survived, and no animals were excluded. There were no procedure-related complications (e.g., gavage injury). At the endpoint, all mice were euthanized for sample collection. Serum, lymph nodes, spleen, and kidney tissues were harvested for subsequent analysis. All procedures were approved by the Ethics Committee of Inner Mongolia Medical University (Approval No. YKD202405055).

### 2.3 LPS-induced THP-1 cell assay

#### 2.3.1 Human THP-1 monocyte experiments

Human THP-1 monocytes were purchased from the cell bank of the Chinese Academy of Sciences. Cells were cultured in RPMI 1640 medium (Bio-Channel, BC-M-005) supplemented with 10% fetal bovine serum (FBS; Bio-Channel, BC-SE-FBS07) and 1% penicillin–streptomycin at 37°C in a humidified incubator with 5% CO_2_. To establish an *in vitro* inflammation model, THP-1 cells were first differentiated into M0 macrophages by treatment with PMA (100 ng/ml; MCE, HY-18739) for 24 hours. Subsequently, the differentiated macrophages were subjected to genetic manipulation via plasmid transfection to establish the desired perturbation. Following transfection and an appropriate recovery period, cells were stimulated with LPS (100 ng/ml; MCE, HY-D1056) or with BG (provided by Professor Su Xiulan, Clinical Medical Research Center, Affiliated Hospital of Inner Mongolia Medical University) at two concentrations (5 μg/ml and 10 μg/ml) for 24 hours to induce inflammatory activation. Cell viability and phenotypic analyses were performed following this treatment schedule.

#### 2.3.2 Mouse splenocyte experiments

Spleens were aseptically harvested from mice and mechanically dissociated into small fragments. The fragments were digested in 5 mL of Hanks’ Balanced Salt Solution (HBSS; Solarbio, H1025) containing 100 U/mL type IV collagenase (Gibco, 17104019) and 20 μg/mL DNase I (Bio-Channel) with 1% FBS at 37°C for 30 min. The digest was then filtered through a 70-μm cell strainer and centrifuged. Erythrocytes were lysed using a red blood cell (RBC) lysis buffer (Solarbio, R1010). After washing, the isolated splenocytes were resuspended in complete RPMI-1640 medium. Cells were adjusted to a density of (2–3) × 10^6^ cells/mL and then treated directly with BG (5 μg/mL or 10 μg/mL) for 24 h.

### 2.4 Cell transfection

For transfection experiments, THP-1 cells were first differentiated into macrophage-like cells using PMA and primed with LPS as described in Section 2.3. miRNA-146a mimics, inhibitors, mimic NC and inhibitor NC, pcDNA-BRD4 and pcDNA-NC were synthesized by Changsha Zebra Biotechnology Co., Ltd. (Changsha, China). The cells were transfected with 25–50 nM miRNA-146a mimic, mimic-NC, miRNA-146a inhibitor and inhibitor NC using LipoHigh liposome efficient transfection reagent (Sangon Biotech).

### 2.5 CCK-8

THP-1-derived macrophage-like cells (THP-1 cells) were seeded into 96-well plates at a volume of 200 μL per well, cultured in a 5% CO_2_ incubator at 37°C for 12–24 h, and treated with LPS or BG for 24 h. Then, 10 μL of CCK8 solution was added to each well and cultured for 4 h. The absorbance of each well at 490 nm was measured on a microplate reader to calculate the relative viability of the cells.

### 2.6 ELISA

The expression of TNF-α, IL-6 and IL-1β in the supernatant of THP-1-derived macrophage-like cells (THP-1 cells) was detected using human TNF-α ELISA kit (Proteintech, KE00154), human IL-6 ELISA kit (Proteintech, KE00139) and human IL-1β-ELISA kit (Proteintech, KE00021). The expression of anti-nuclear antibody (ANA) (Antibodies, ABIN366290), anti-double-stranded DNA antibody (anti-dsDNA) (Antibodies, ABIN366291), and inflammatory factors in mouse serum was detected using mouse ELISA kit (Elabscience, E-MSEL-M0002, E-EL-M0037c, E-EL-M0044c). Each experiment was repeated at least three times.

### 2.7 Quantitative real-time

In the RT-PCR analysis, total RNA was extracted using a kit (Sangon Biotech, B518811) and reverse transcribed using the First Strand cDNA Synthesis Kit (GBTbiotech, P118-100) according to the instructions of manufacturer. The primers used in this study are as follows: GAPDH (forward sequence (F): 5’-GACATCCGATAAAATTGGAACG-3’ and reverse sequence (R): 5’-TTGGACCATTTCTCGATTTGTG-3’), U6 (F: 5’-GACATCCGATAAAATTGGAACG-3’ and R: 5’-TTGGACCATTTCTCGATTTGTG-3’), miR-146a (F: 5’-CCGATGTGTATCCTCAGCTTTG-3’ and R: 5’-TCCCAGCTGAAGAACTGAATTT-3’), BRD4 (F: 5’-CTAGCGTCTCAGAGTGCCTG-3’ and R: 5’-TGCCTCTTGGGCTTGTTAGG-3’). GAPDH and U6 were used as negative control. Finally, the relative expression was calculated using the 2^−ΔΔct^ method.

### 2.8 Western blot

THP-1-derived macrophage-like cells (THP-1 cells) were lysed with PMSF-containing lysates (Beijing Dingguo Changsheng Company) and protein concentrations were determined using a BCA kit (Beijing Dingguo Changsheng Company). The appropriate SDS PAGE gel was selected according to the molecular size of the protein to be detected, and gel electrophoresis was performed according to the extracted protein concentration. LC3B and SQSTM1 (Sequestosome 1) are two important marker proteins in the detection of autophagy. The changes in LC3B reflect the process of autophagosome formation, while the degradation of SQSTM1 indicates the activity of the autolysosome pathway. By monitoring these two proteins simultaneously, a more comprehensive assessment of the normality and regulatory mechanisms of cellular autophagic function can be achieved. Therefore, WB was performed using specific antibodies against LC3B (proteintech, 18725-1-AP, 1:2500), SQSTM1 (proteintech, 18420-1-AP, 1:10000), BRD4 (proteintech, 28486-1-AP, 1:2000), and β-actin (proteintech, 81115-1-RR, 1:20000). ChemiScope 6100 was used as imaging system.

#### 2.8.1 Quantification and data normalization

Band intensities were quantified using ImageJ software (National Institutes of Health). The expression level of each target protein (LC3B-II, SQSTM1, BRD4) is presented as its band intensity normalized to that of the loading control (β-actin) from the same sample. No further normalization across experimental groups was performed. Statistical analyses were conducted on these normalized intensity ratios.

### 2.9 Immunofluorescence

THP-1-derived macrophage-like cells (THP-1 cells) were permeabilized with 0.1% Triton 100 for 15 min, incubated overnight with primary antibody. After washing with PBS and blocking with 5%BSA were incubated with secondary antibody at 37°C for 1 h. The primary antibody was rabbit anti-LC3B (proteintech, 18725-1-AP), SQSTM1 (proteintech, 66184-1-lg), BRD4 (proteintech, 28486-1-AP), and the secondary antibody was Goat anti-Mouse/Rabbit Poly-HRP (proteintech, PR30009). The kidneys and spleens of mice were subjected to antigen repair after paraffin sections and primary antibodies were added overnight at 4°C, and then secondary antibodies were added to incubate for 50 min at room temperature in the dark. Primary antibodies included F4/80 antibody (Affinity Biosciences, 29414-1-AP, 1:200), CD86 antibody (Affinity Biosciences, #DF6332, 1:500), CD206 antibody (Affinity Biosciences, #DF4149, 1:500) and LC3B antibody (Affinity Biosciences, #AF4650, 1:500). Fluorescent secondary antibodies included FITC-labeled goat anti-rabbit IgG (Servicebio, GB22303, 1:400), FITC-labeled goat anti-mouse IgG (Servicebio, GB22301, 1:400), Cy3-labeled goat anti-rabbit IgG (Servicebio, GB21303, 1:400) and Cy3-labeled goat anti-mouse IgG (Servicebio, GB21301, 1:400). The cell nuclei were stained by 4’,6-diamidino-2 phenylindole (DAPI; Solarbio, C0060).

### 2.10 Transmission electron microscope

THP-1-derived macrophage-like cells (THP-1 cells) were seeded into 24-well plates and cultured for 24 h. The cells were collected and fixed with 4% glutaraldehyde (Solarbio, P1126) for 12 h, then fixed with 1% osmium tetroxide (Shanghai Rongbai Biology, RBS0086) for 2 h, dehydrated with gradient ethanol, embedded with epoxy resin, and then made into 50–100nm ultrathin sections. Finally, the sections were stained with 3% uranyl acetate and lead citrate. The autophagosomes of cells were observed under transmission electron microscope (HT7800 (80KV)).

### 2.11 Flow cytometry

THP-1-derived macrophage-like cells (THP-1 cells) were seeded and treated in 6-well plates. The cells were collected, washed and blocked with FBS for 10-15min. PE anti-mouse CD206 antibody (Biolegend, 141705), APC anti-mouse CD11b antibody (Biolegend, 101211) and FITC anti-mouse CD86 antibody (Biolegend, 105005) were incubated on ice in the dark for 15 min. The proportion of cells was analyzed by flow cytometry (BD FACSCanto) within 1 h.

### 2.12 Dual-luciferase reporter assay

THP-1-derived macrophage-like cells (THP-1 cells) were divided into the following four groups: BRD4-WT+mimic NC, BRD4-WT+miR-146a, BRD4-MUT+mimic NC, BRD4-MUT+miR-146a. The cells were seeded in 96-well plates and cultured at 37°C and 5% CO_2_ to a density of 95%. Subsequently, the BRD4 overexpression vector was transfected with transfection reagents (Gene Pharma, 220721). The detection was performed using a dual luciferase assay kit (Beyotime, RG021S).

### 2.13 Immunohistochemistry

Formalin-fixed paraffin-embedded tissue sections were removed in xylene, rehydrated by graded ethanol, and then placed in a dye vat for hematoxylin-eosin (HE) staining (Pinofide Biotechnology Co., S191003). After staining with HE staining solution I for 3–5 min, the sections were washed with stained cup water to colorless, and then stained with HE staining solution II and III for 3–5 s, and quickly washed with water. Finally, each cylinder was immersed in 85% ethanol, 95% ethanol, HE staining solution IV, anhydrous ethanol I, anhydrous ethanol II, anhydrous ethanol III, n-butanol, xylene I, xylene II for 3–5 min. After staining, the slices were taken out and put into tuyere for drying. Slices were sealed with neutral gum and observed under a microscope.

### 2.14 Colorimetry

Proteinuria in mice was measured by Elabscience^®^ urine protein colorimetric test kit (Elabscience, E-BC-K252-M). The concentration of blood urea nitrogen in mice was detected by Elabscience^®^ urea (BUN) colorimetric test kit (Elabscience, E-BC-K183-M). The concentration of serum creatinine in mice was detected by creatinine (Cr) assay kit (Nanjing Jiancheng Bioengineering Institute, C011-2-1).

### 2.15 Statistical analysis

All experiments were performed at least three times. The results are expressed as mean ± SD. SPSS method was used for statistical analysis. The normality of data distribution was assessed using the Shapiro-Wilk test, and homogeneity of variances was assessed using Levene’s test. For comparisons between two groups, an unpaired two-tailed Student’s t-test was used for normally distributed data; otherwise, the Mann-Whitney U test was applied. For comparisons among multiple groups, one-way ANOVA followed by Tukey’s *post hoc* test was used for parametric data, and the Kruskal-Wallis test with Dunn’s *post hoc* test was used for non-parametric data. A P value of less than 0.05 was considered statistically significant.

## 3 Results

### 3.1 BG alleviated disease in lupus mice

**Figure 1:**
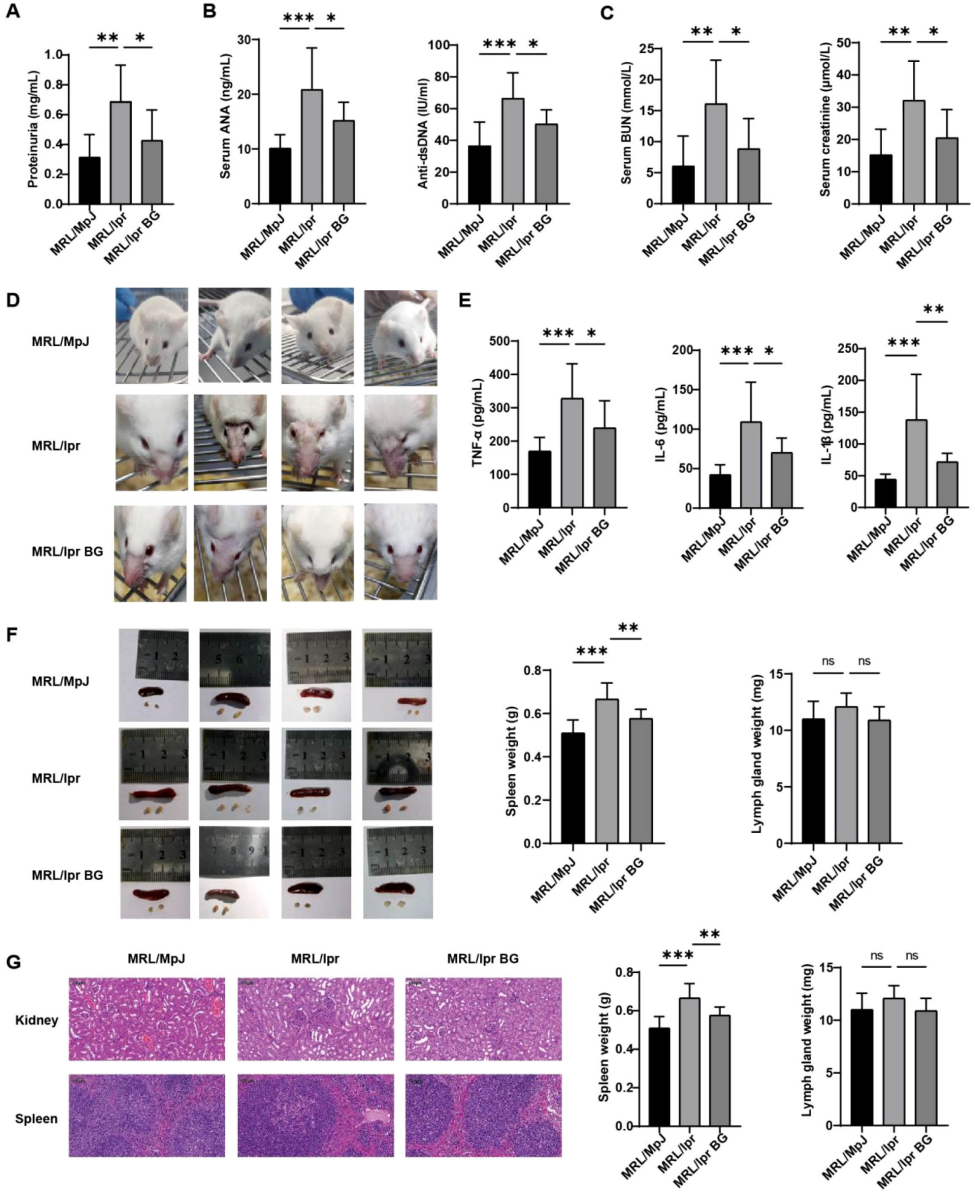
BG treatment alleviated lupus in mice. **(A)** proteinuria, **(B)** antinuclear antibodies (ANA) and anti-dsDNA antibodies **(C)** creatinine and blood urea nitrogen in serum of mice in each group. **(D)** Skin lesions of mice in each group. **(E)** Serum concentrations of inflammatory factors TNF-α, IL-6 and IL-1β measured in each group. **(F)** Size and weight of spleen and lymph nodes in each group of mice. **(G)** H&E histology of kidney and spleen of mice in each group. *P < 0.05, **P < 0.01, ***P < 0.001, ns, no statistical significance.

We evaluated the therapeutic effects of BG in lupus-prone MRL/lpr mice. BG treatment significantly reduced proteinuria, serum levels of antinuclear antibodies (ANA), anti-dsDNA antibodies, blood urea nitrogen, and creatinine compared to untreated MRL/lpr controls (**Figure 1A–C**). It also alleviated the extension of cutaneous lesions (**Figure 1D**) and lowered serum levels of the pro-inflammatory cytokines TNF-α, IL-6, and IL-1β (**Figure 1E**). Furthermore, BG treatment prevented spleen enlargement, while no significant change was observed in lymph node size (**Figure 1F**). Histological analysis of kidney and spleen tissues revealed severe pathology in MRL/lpr mice, including glomerular enlargement, mesangial and endothelial cell proliferation, inflammatory cell infiltration, tubular edema with loss of brush border in kidneys, as well as expanded lymphoid follicles, diffuse white pulp proliferation, increased germinal centers, and red pulp inflammation in spleens. BG treatment markedly reduced these inflammatory and pathological changes in both organs (**Figure 1G**). No significant difference in survival rate was observed among the groups during the study period. These results demonstrate that BG alleviates key clinical, serological, and histopathological features of lupus in MRL/lpr mice.

### 3.2 BG promote M2 macrophage polarization

**Figure 2:**
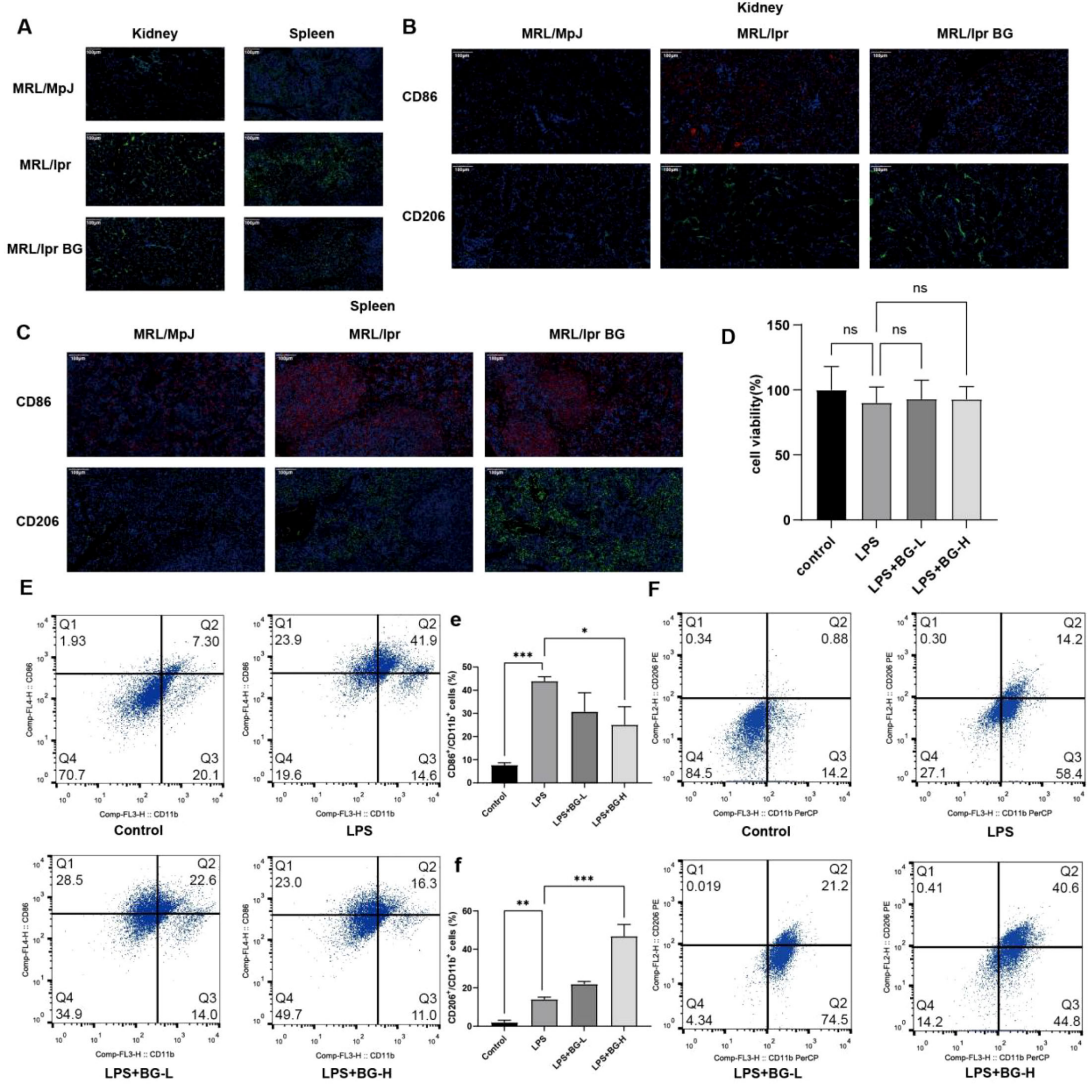
Regulation of BG on macrophage polarization. **(A)** Expression of F4/80 in kidney/spleen of mice in each group (n=6). **(B)** Expression of CD86 and CD206 in kidney of mice in each group (n=6). **(C)** CD86 and CD206 expression in spleen of mice in each group (n=6). **(D)** CCK8 assay used to detect cell viability in each group. **(E)** Proportion of M1 (CD86/CD11b) macrophages detected by flow cytometry. **(F)** Proportion of M2 (CD206/CD11b) macrophages detected by flow cytometry. *P < 0.05, **P < 0.01, ***P < 0.001, ns, no statistical significance.

The pathogenesis of SLE is usually accompanied by infiltration of immune cells, including macrophages. Using immunofluorescence, we found that F4/80 macrophages infiltrated in the kidney and spleen of MRL/lpr mice. Macrophage infiltration was significantly reduced after BG treatment (**Figure 2A**). Further detection of macrophage subtypes by immunofluorescence showed an increase in CD86^+^CD206^+^ cells in kidney and spleen from MRL/lpr mice, while BG treatment decreased CD86^+^ cells and increased CD206^+^ cells (**Figures 2B, C**). To test the effect of BG on macrophage polarization *in vitro*, we established an LPS-induced macrophage model. First, we determined the effect of LPS and BG at low and high concentrations on THP-1 cell viability using the CCK-8 assay. We found that treatment with LPS and low and high concentrations of BG had no effect on cell viability (**Figure 2D**). We validated these findings by flow cytometry, we found that the proportion of CD86/CD11b-positive and CD206/CD11b-positive cells were both significantly increased after LPS induction, while the proportion of CD86/CD11b-positive cells decreased, and the proportion of CD206/CD11b-positive cells increased after BG treatment (**Figures 2E, F**). These results suggests that BG treatment inhibits macrophage M1 polarization and promotes M2 polarization.

### 3.3 BG modulates autophagy markers in tissues and promotes autophagic activity in macrophages

Autophagy is a key physiological pathway for maintaining cellular homeostasis. To investigate whether BG influences autophagy, particularly within the immune context of lupus, we performed a multi-level analysis. First, at the tissue level, Western blot analysis of whole kidney and spleen lysates revealed an upregulation of the autophagy marker LC3B-II and a downregulation of the autophagy substrate SQSTM1/p62 in MRL/lpr mice compared to healthy controls. BG treatment further enhanced these changes (**Supplementary Figure 1**). These data indicate that BG induces a general activation of autophagic flux in lupus-affected tissues, although the cellular source of these markers cannot be specified from whole-tissue extracts.

**Figure 3:**
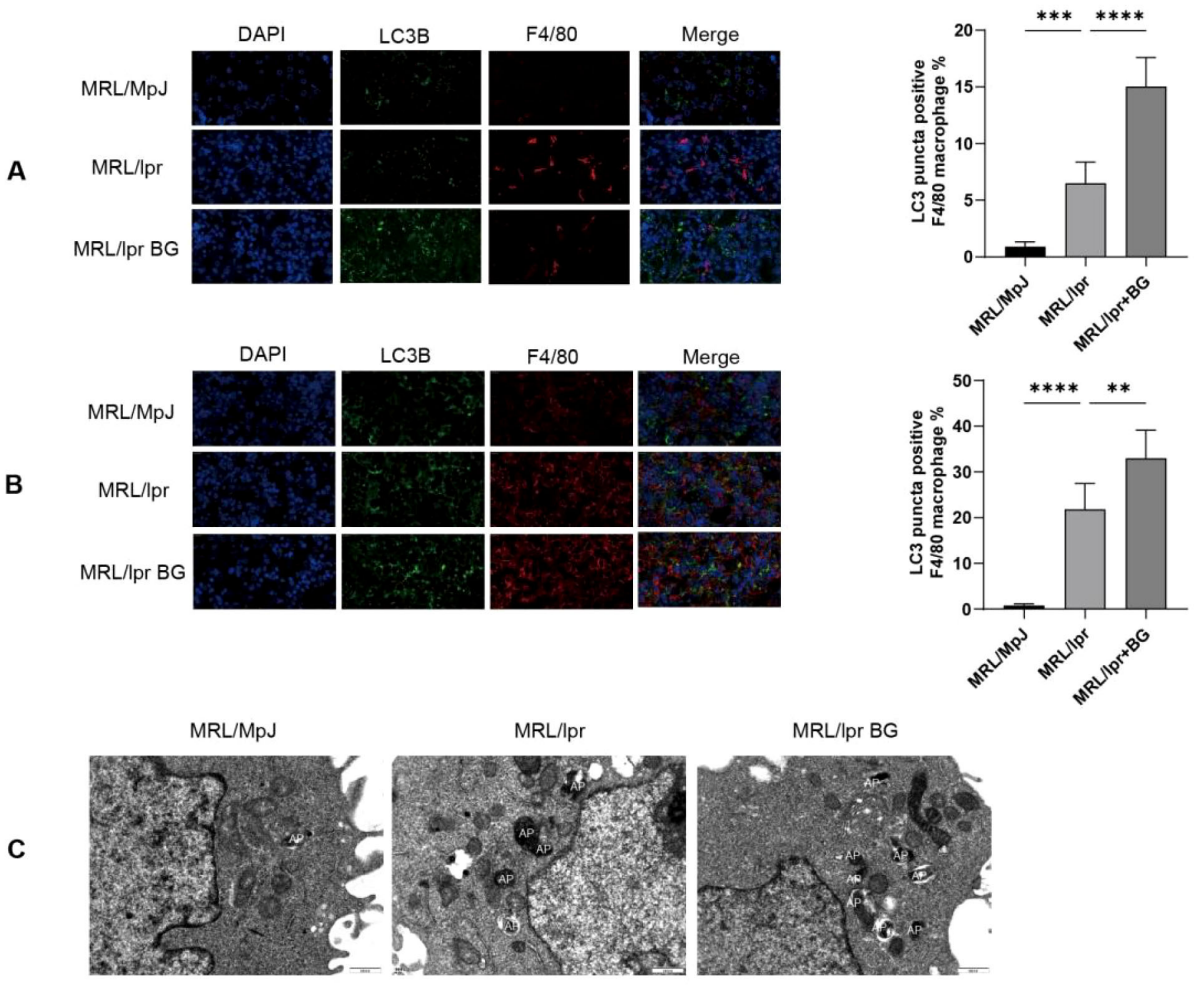
BG regulates macrophage autophagy. **(A)** Expression of LC3B/F4/80 in kidney of mice in each group. **(B)** Expression of LC3B/F4/80 in spleen of mice in each group. **(C)** Number of autophagosomes in kidney tissues of mice in each group detected by electron microscopy. *P < 0.05, **P < 0.01, *** P<0.005, **** P<0.001.

To specifically assess autophagy within macrophages, we performed immunofluorescence co-staining for LC3B and the macrophage marker F4/80. In the kidneys and spleens of MRL/lpr mice, we observed an increase in LC3B+F4/80+ cells. Importantly, BG treatment significantly increased the proportion of these double-positive cells (**Figures 3A, B**), suggesting an enhancement of autophagic activity specifically within tissue-resident macrophages.

This finding was corroborated by transmission electron microscopy (TEM). Ultrastructural analysis of kidney tissues showed an increased number of autophagosomes in MRL/lpr mice, which was further elevated upon BG treatment (**Figure 3C**). Together, the immunofluorescence and TEM data provide direct visual evidence that BG promotes autophagic activity *in vivo*, with a clear effect observed within macrophages.

*In vitro* experiments using LPS-primed human THP-1-derived macrophages yielded consistent results. BG treatment increased the expression of LC3B and decreased SQSTM1/p62 (**Supplementary Figures 2**-**4**). Most conclusively, TEM quantification confirmed a significant increase in autophagosome numbers in BG-treated cells compared to LPS controls (**Supplementary Figure 5**). These *in vitro* results confirm that BG can directly enhance autophagic flux in human macrophages under inflammatory conditions.

### 3.4 BG inhibits macrophage inflammatory response through induction of miR-146a

**Figure 4:**
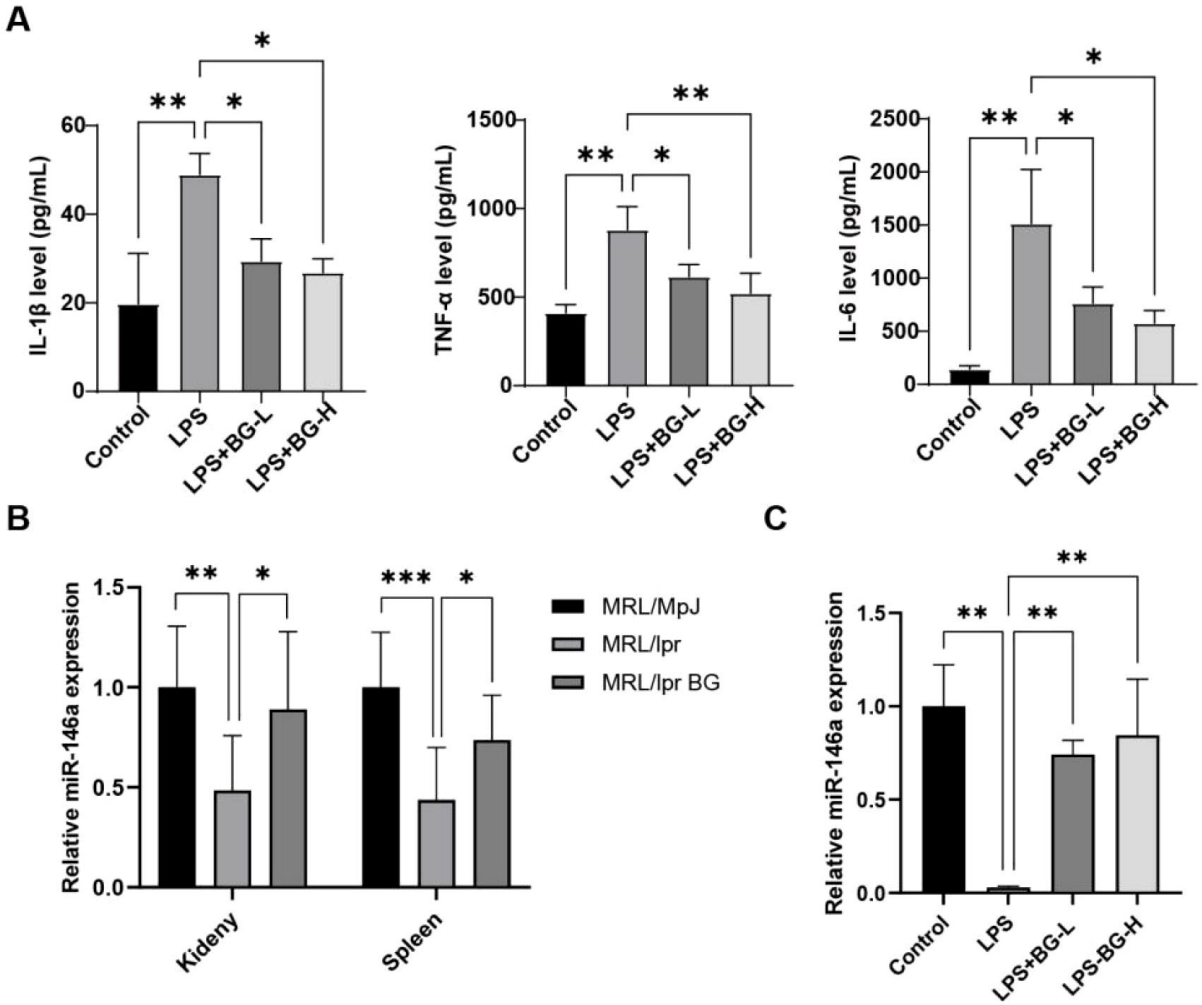
BG inhibits macrophage inflammatory response through induction of miR-146a. **(A)** ELISA used to detect inflammatory factors IL-1β, TNF-α and IL-6 in supernatants of THP-1 cell (THP-1-derived macrophage-like cells) cultures. **(B)** Expression of miR-146a in kidney/spleen of mice in each group. **(C)** Expression of miR-146a in THP-1 cells (THP-1-derived macrophage-like cells) after treatment in each group. *P < 0.05, **P < 0.01, ***P < 0.001.

M1 macrophages participate to the inflammatory response in SLE. Thus, we used the LPS-induced THP-1 cell assay to examine the effect of BG on pro-inflammatory cytokine production *in vitro*. The levels of inflammatory cytokines IL-1β, TNF-α and IL-6 were significantly decreased upon BG treatment (*P* < 0.05) (**Figure 4A**). This indicated that BG inhibits the inflammatory response of THP-1 cells induced by LPS. miR-146a is an inhibitor of the classic NF-κB pro-inflammatory pathway. Therefore, qPCR was employed to detect expression of miR-146a in mouse kidney, spleen and LPS-induced THP-1 cells. BG treatment could upregulate the expression of miR-146a in kidney and spleen of MRL/lpr mice and LPS-induced THP-1 cells (**Figure 4B, C**).

### 3.5 Effect of miR-146a on LPS-induced M1 macrophages

To investigate the role of miR-146a in macrophage polarization and autophagy under inflammatory conditions, we performed gain-of-function experiments in the established LPS-primed, THP-1-derived macrophage model. Briefly, cells were first transfected with miR-146a mimics or a negative control, then differentiated with PMA and subsequently stimulated with LPS as described in the Methods.

**Figure 5:**
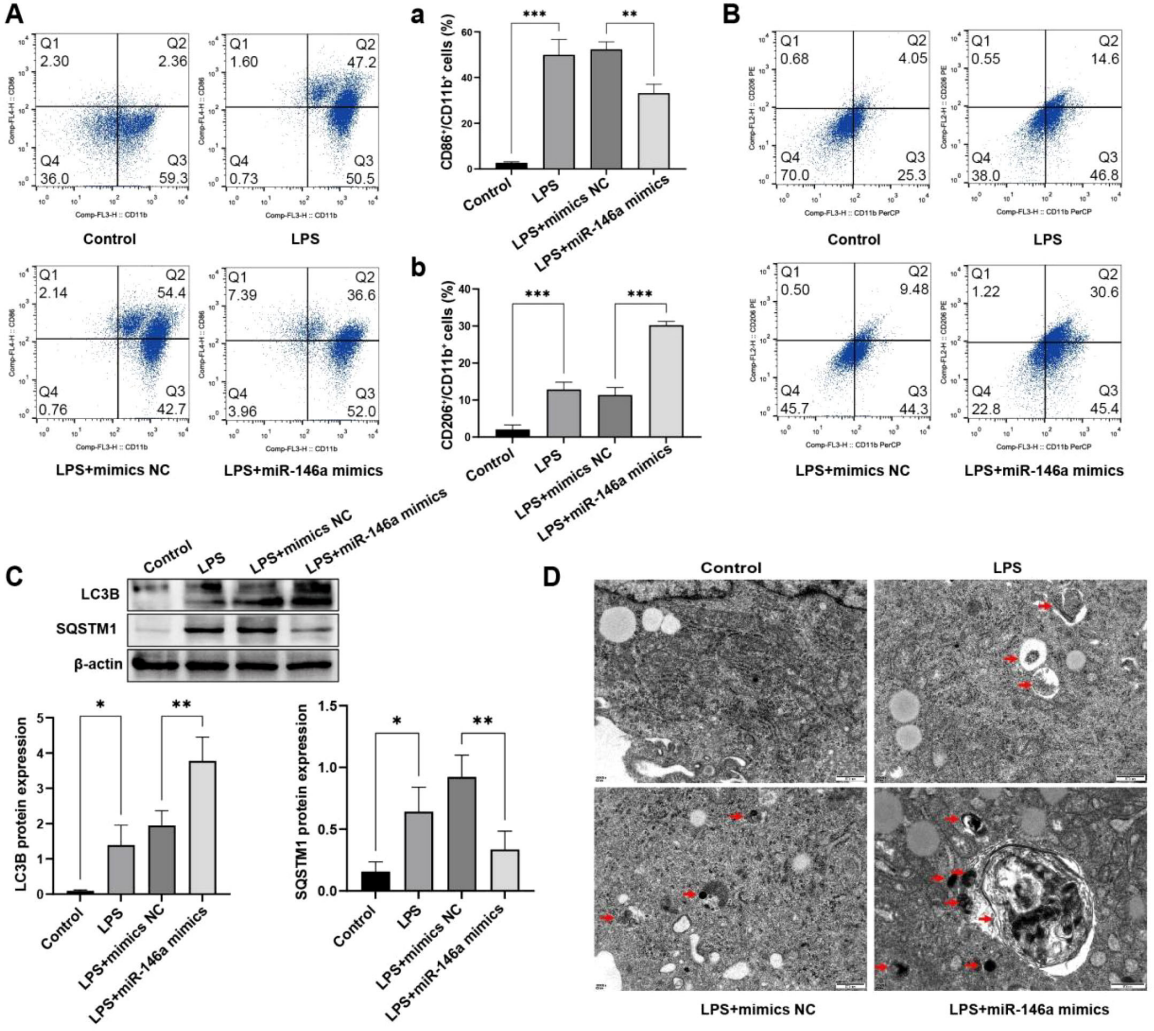
The effect of miR-146a on LPS-induced M1 macrophages. **(A)** Proportion of M1 (CD86/CD11b) macrophages in each group. **(B)** Proportion of M2 (CD206/CD11b) macrophages in each group. **(C)** Expression of autophagy-related proteins in each group detected by WB. **(D)** Number of autophagosomes in each group detected by electron microscopy. *P < 0.05, **P < 0.01, ***P < 0.001.

Successful transfection was confirmed by qPCR, which showed a significant upregulation of miR-146a expression in the mimic group compared to the control (**Supplementary Figure 6**). Flow cytometry analysis of surface markers revealed that overexpression of miR-146a significantly decreased the proportion of CD86+CD11b+ cells while increasing the proportion of CD206+CD11b+ cells (**Figures 5A, B**). This shift in marker expression suggests that miR-146a can attenuate LPS-induced M1-like polarization and promote an M2-like phenotype.

We next examined the effect of miR-146a on autophagy in this model. Western blot analysis showed that miR-146a overexpression further increased the LPS-induced level of LC3B-II and decreased the level of SQSTM1/p62 (**Figure 5C**; quantified in **Supplementary Figure 7**). This pattern was corroborated by immunofluorescence (**Supplementary Figure 8**). Consistently, transmission electron microscopy (TEM) demonstrated a significant increase in the number of autophagosomes in miR-146a-overexpressing macrophages compared to LPS-treated controls (**Figure 5D**). These combined data indicate that miR-146a enhances autophagic activity in LPS-primed macrophages.

### 3.6 BG regulates macrophage polarization and autophagy by up-regulating miR-146a

**Figure 6:**
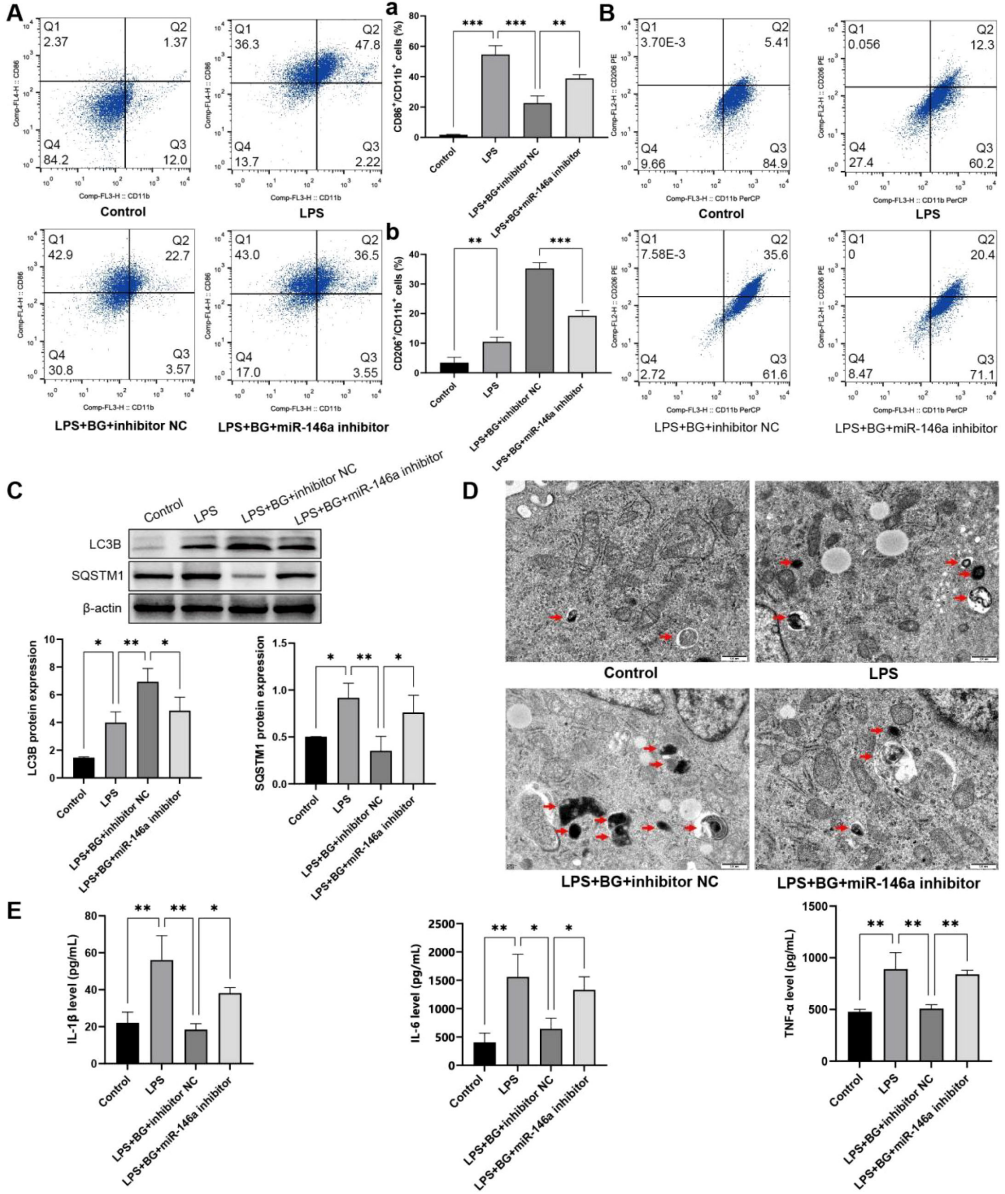
The miR-146a inhibitor reversed the regulation of BG on macrophage polarization and autophagy. **(A)** Proportion of M1 (CD86/CD11b) macrophages in each group. **(B)** Proportion of M2 (CD206/CD11b) macrophages in each group. **(C)** Expression of autophagy-related proteins in each group detected by WB. **(D)** Number of autophagosomes in each group detected by electron microscopy. **(E)** Levels of inflammatory factors in each group detected by ELISA. *P < 0.05, **P < 0.01, ***P < 0.001.

To assess whether miR-146a was involved in the effect of BG on LPS-induced M1-type macrophages, we transfected THP-1 cells with miR-146a inhibitor and induced differentiation with PMA and LPS. The results of qPCR showed that miR-146a inhibitors inhibited the up-regulation of miR-146a expression by BG (*P* < 0.05) (**Supplementary Figure 9**). Flow cytometry results revealed that miR-146a inhibitors inhibit the down-regulation of BG on the proportion of CD86/CD11b positive cells and the up-regulation of CD206/CD11b positive cells (**Figures 6A, B**). The results of WB and immunofluorescence showed that miR-146a inhibitors inhibited the up-regulation of LC3B expression and the down-regulation of SQSTM1 expression by BG (**Figure 6C**; **Supplementary Figure 10**, **11**). Furthermore, transmission electron microscopy revealed that miR-146a inhibitors could reduce the number of autophagosomes increased by BG (**Figure 6D**). In addition, we also measured the expression of inflammatory factors and found that miR-146a inhibitors inhibited the down-regulation of BG on IL-1β, TNF-α, and IL-6 levels (*P* < 0.05) (**Figure 6E**). Collectively, the above findings suggest that miR-146a is an important regulator of macrophage activity by BG. BG promotes M1 polarization to inhibit M2 polarization and activate autophagy by promoting miR-146a expression. In addition, BG inhibited the inflammatory response of M1-type macrophages by promoting miR-146a expression.

### 3.7 miR-146a targets BRD4 and inhibits its expression

**Figure 7:**
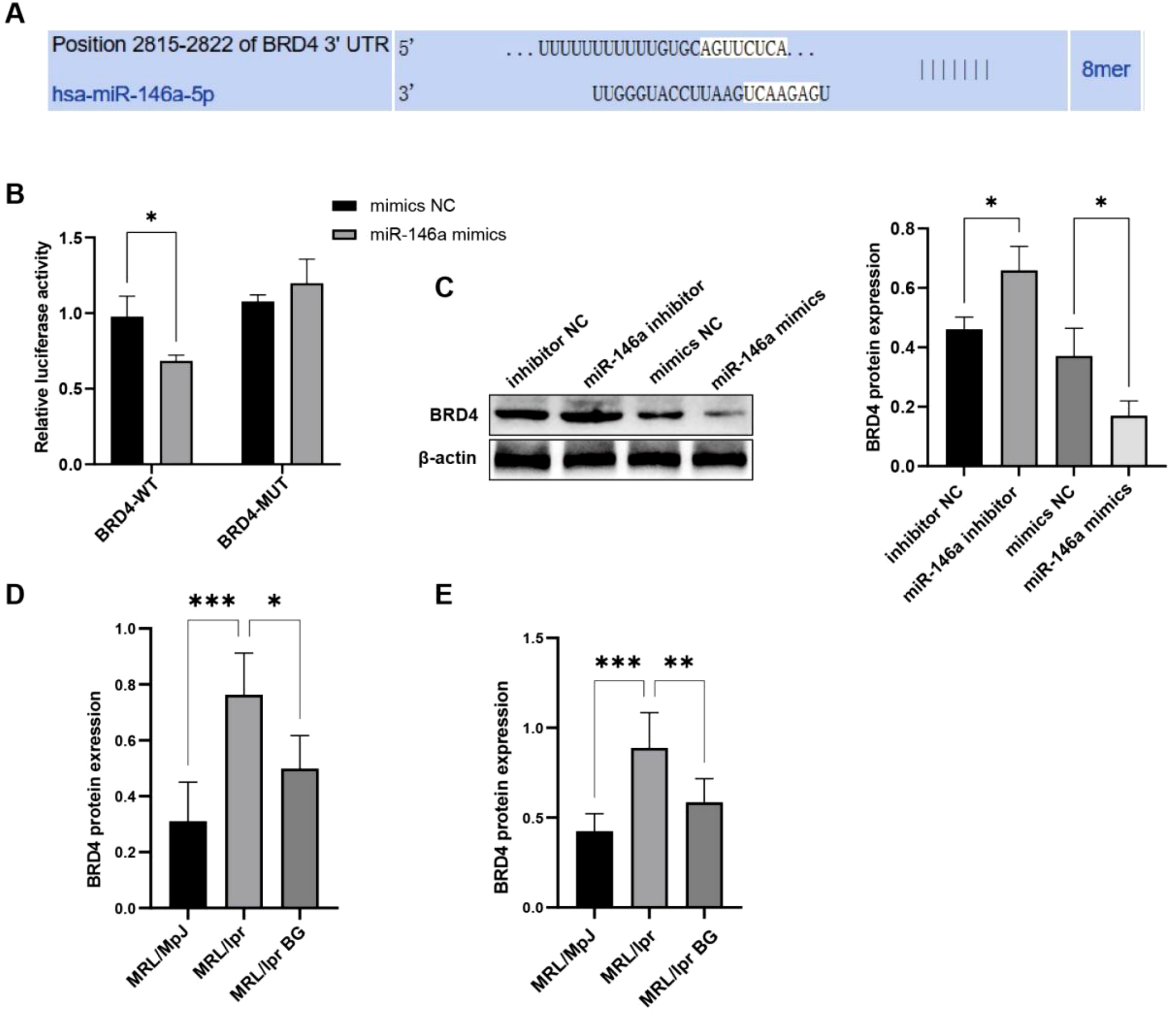
miR-146a targets BRD4 and inhibits its expression. **(A)** Targetscan predicts miR-146a binding sites. **(B)** Dual luciferase assay verified the targeting of miR-146a and BRD4. **(C)** WB used to detect the expression of BRD4. **(D)** Expression of BRD4 in kidney tissues of mice in each group. **(E)** Expression of BRD4 in spleen tissues of mice in each group. *P < 0.05, **P < 0.01, ***P < 0.001.

To elucidate the mechanism by which BG regulates macrophage activity, we identified a binding site between miR-146a and BRD4 using miRNA binding site prediction software TargetScan (**Figure 7A**). Next, we used a luciferase reporter gene assay to verify the targeted binding of miR-146a and BRD4 in THP-1 cells. The results demonstrated that fluorescence activity of the BRD4-WT+miR-146a mimic group was significantly lower than that of the BRD4-WT+miR-146a mimic NC group (*P* < 0.05). There was no significant difference in the fluorescence activity between the BRD4-MUT+miR-146a mimic NC group and the BRD4-MUT+miR-146a mimic group (**Figure 7B**). This indicated a targeting relationship between miR-146a and BRD4. Western blot and immunofluorescence results confirmed that overexpression of miR-146a inhibited BRD4 expression (**Figure 7C**; **Supplementary Figure 12**). In addition, we detected expression of BRD4 in kidney and spleen of untreated lupus mice and found that BG treatment downregulated this expression (**Figures 7D, E**).

### 3.8 BRD4 inhibits the regulation of BG on macrophages

**Figure 8:**
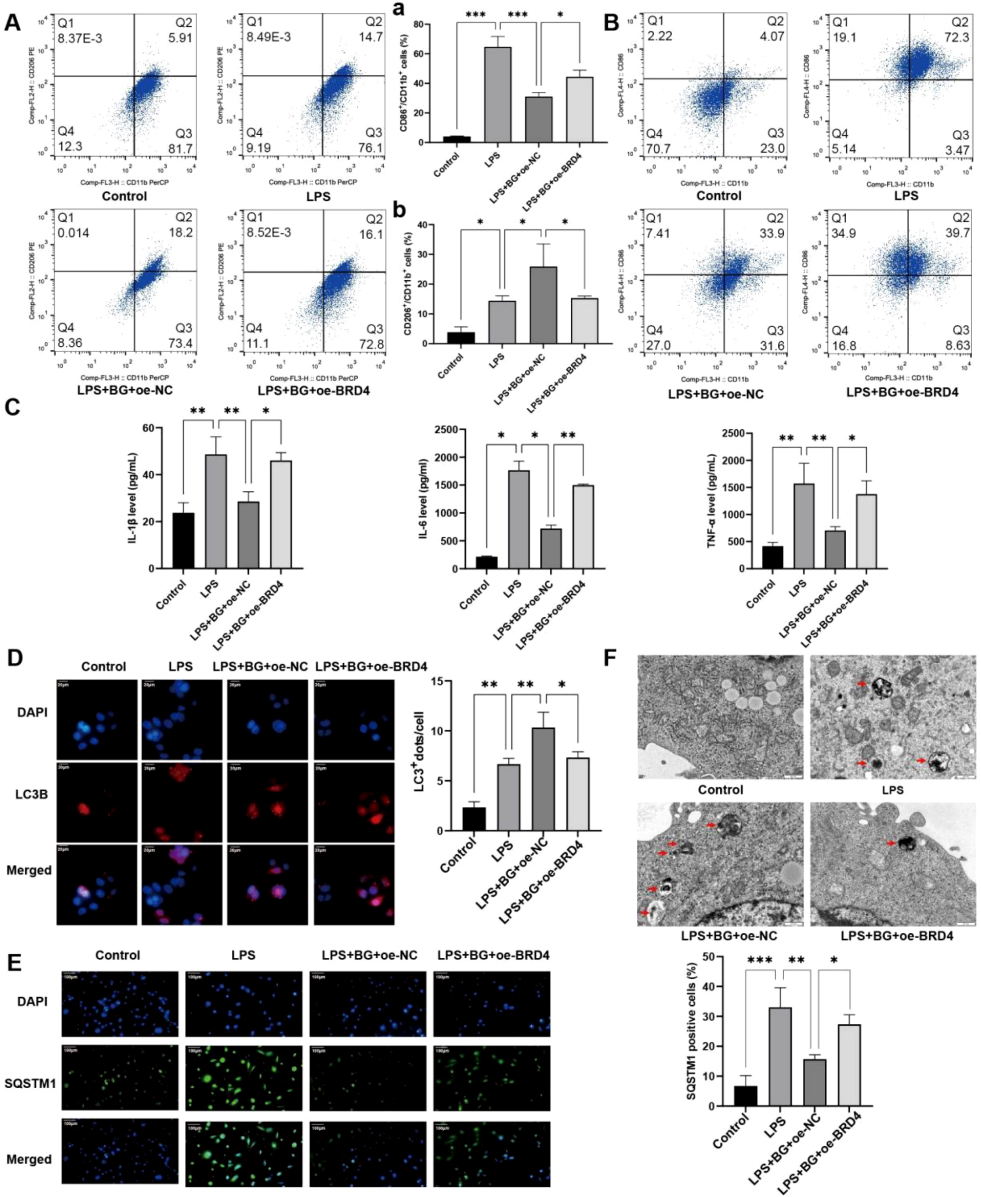
BRD4 inhibits the regulation of BG on macrophages. **(A)** Proportion of M1 (CD86/CD11b) macrophages in each group. **(B)** Proportion of M2 (CD206/CD11b) macrophages in each group. **(C)** Levels of inflammatory factors in each group detected by ELISA. **(D)** Expression of LC3B in each group detected by immunofluorescence. **(E)** Expression of SQSTM1 in each group detected by immunofluorescence. **(F)** Number of autophagosomes in each group detected by transmission electron microscopy. *P < 0.05, **P < 0.01, ***P < 0.001.

Our study demonstrated that BRD4 is a target of miR-146a regulation. However, the role of BRD4 in the regulation of BG on macrophages remains to be elucidated. First, we detected expression of BRD4 by immunofluorescence. LPS significantly upregulated BRD4 expression, and BG treatment downregulated BRD4 expression in LPS-induced cells (**Supplementary Figure 12**). Our results showed that overexpression of BRD4 partially reversed the effect of BG on LPS-induced macrophages. It decreased the proportion of M1 macrophages and increased the proportion of M2 macrophages (**Figures 8A, B**). Furthermore, BRD4 significantly elevated the concentration of inflammatory factors that were reduced in presence of BG (**Figure 8C**). Further detection of autophagy-related proteins revealed that LC3B was upregulated and SQSTM1 was downregulated after BG treatment, indicating that BG activated autophagy, whereas overexpression of BRD4 reversed the activation of autophagy by BG (**Figures 8D, E**; **Supplementary Figure 13**). Transmission electron microscopy results confirmed that BRD4 inhibited the increased number of autophagosomes caused by BG (**Figure 8F**).

### 3.9 BG inhibits splenocytes activation in lupus mice

**Figure 9:**
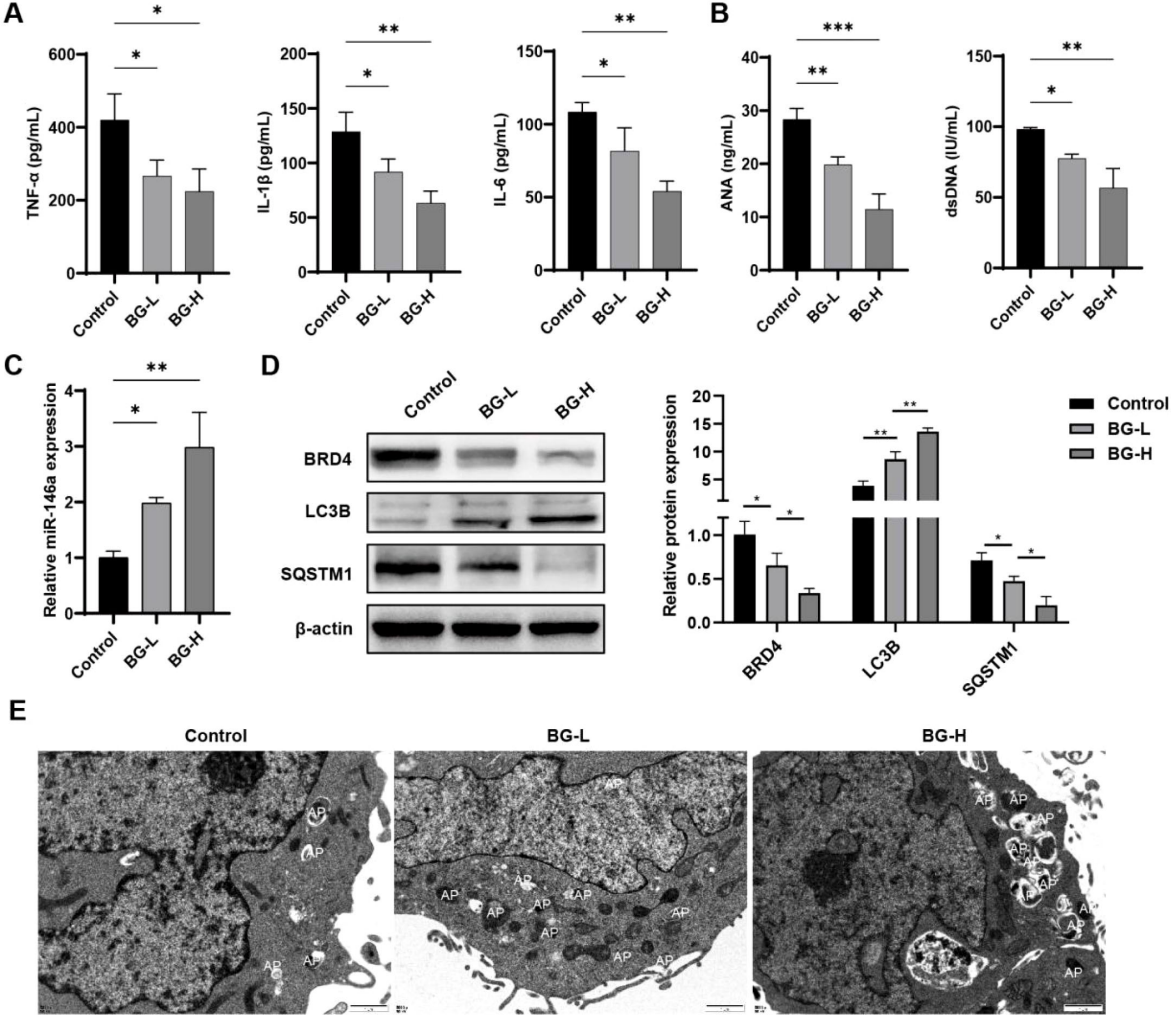
BG decreases inflammation of primary splenocytes of lupus mice. **(A)** Levels of TNF-α, IL-6 and IL-1β in the supernatant of primary splenocyte culture. **(B)** Levels of ANA and anti-dsDNA antibody in the supernatant of primary splenocyte culture. **(C)** Expression of miR-146a in the supernatant of cultured primary splenocytes. **(D)** Expression of BRD4/LC3B/SQSTM1 in primary splenocytes. **(E)** Number of autophagosomes in primary spleen cells detected by transmission electron microscopy. *P < 0.05, **P < 0.01, ***P < 0.001.

Splenocytes were obtained from MRL/lpr mice and subjected to functional studies. We found that BG reduced levels of TNF-α, IL-6, and IL-1β (*P* < 0.05, *P* < 0.01), levels of Antinuclear antibodies (ANAs) and anti-dsDNA antibodies and increased miR-146a expression (*P* < 0.01) in a dose-dependent manner (**Figures 9A-C**). BG treatment downregulated expression of Bromodomain-containing protein 4 (BRD4) and SQSTM1 in splenocytes of MRL/lpr mice, and upregulated expression of LC3B (*P* < 0.05. P < 0.01) (**Figure 9D**). In addition, we observed under electron microscope that BG treatment promoted the formation of autophagosomes in spleen cells of MRL/lpr mice (**Figure 9E**). These results suggest that BG inhibits the inflammatory response of spleen cells and activates autophagy.

## 4 Discussion

Bioactive peptides are small molecules, composed of 2–20 amino acids, with of anti-oxidation, anti-hypertension, anti-diabetes, anti-inflammation and anti-cancer activities. At present, bioactive peptides have been shown to have promising curative effects in many diseases. Our previous studies have shown that bioactive peptides can inhibit THP-1 cells proliferation, migration and invasion and induce apoptosis by regulating RNA modifications and targeting the Wnt/β-catenin signaling pathway (19). Ginseng oligopeptides regulate innate immune response by enhancing macrophage phagocytosis and NK cell activity (20). Using a model of ulcerative colitis, we found that bioactive peptides can regulate the balance of regulatory T cells/helper T cells 17 (Treg/Th17) and inhibit the activation of TLR4/NF-κB signaling pathway. The therapeutic potential of bioactive peptides is of great importance in prevention and treatment of diseases. The peptide BG used in this study is derived from bitter melon, which has been shown to have anti-diabetic, anti-inflammatory and anti-cancer activities (21). Studies have confirmed that bitter melon extract can inhibit proliferation and induce apoptosis of tumor cells. Our study reports that BG treatment significantly reduces the extension of skin lesions, spleen hypertrophy, kidney damage, and proteinuria in lupus mice. SLE is characterized by presence of abnormally activated T and B lymphocytes and a large number of autoantibodies that form antigen-antibody complexes and deposit in organs such as kidney and spleen resulting in multi-organ damage and eventually death (22). BG treatment reduced serum ANAs and anti-dsDNA antibodies, as well as serum TNF-α, IL-6 and IL-1β concentrations in lupus mice. In addition, BG improved renal function in lupus mice reducing serum creatinine and blood urea nitrogen levels.

Macrophages are the main subset of immune cells infiltrating the kidney and spleen of patients with lupus. They secrete chemokines and cytokines, leading to kidney and spleen inflammation (23, 24). Our results confirmed that F4/80 positive cells accumulate in the kidney and spleen of MRL/lpr mice. BG treatment of MRL/lpr mice inhibited macrophage infiltration. Macrophages respond to environmental cues by differentiating into M1 or M2 subtype of macrophages. Their remarkable plasticity enables them to help eliminate foreign bodies, participate in tissue repair and homeostasis. In non-inflammatory conditions, macrophages can recognize and eliminate apoptotic cells (25). However, in patients diagnosed with SLE, macrophages have reduced cell-clearance activity, leading to accumulation of apoptotic cells, thereby exacerbating pro-inflammatory polarization of macrophages (26). Our study found that BG has a regulatory effect on macrophage polarization, which can inhibit M1 polarization and promote M2 polarization inhibiting inflammatory response. Our findings are in agreement with those reported by Andrea et al. In this study, the authors found that BG-4 isolated from bitter melon inhibited the secretion of pro-inflammatory molecules such as NO, IL-6 and TNF-α by LPS-activated macrophages (27). It has been shown that macrophages in SLE have impaired autophagic degradation capacity (28), which is consistent with our findings. Regarding the effect of BG on macrophage autophagy, we showed that BG promoted autophagy in lupus mouse kidney and spleen cells, LPS-induced THP-1 cells *in vitro*, and mouse primary spleen cells.

To explore the molecular mechanism by which BG promotes macrophage polarization and autophagy, we conducted *in vitro* experiments. miR-146a plays an important role in the pathogenesis of SLE. For example, miR-146a leads to abnormal activation of IFN-I pathway in human lupus by targeting key signaling proteins (29). Studies have shown that the use of exosome-encapsulated delivery of miR-146a-5p promotes the polarization of M1 macrophages to M2 by reducing the expression of NOTCH1, thereby reducing the inflammatory response in lupus mice and reducing SLE-related diffuse alveolar hemorrhage (30). In addition, miR-146a promotes M2 polarization by inhibiting the TLR4/NF-κB axis (31) and activates autophagy by targeting TRAF6 (32). In the present study, we confirmed that inhibition of miR-146a expression reversed the regulatory effect of BG on macrophages. BRD4 is a target of miR-146 and plays an important role in autoimmune and inflammatory diseases. Previous studies suggested that inhibition of BRD4 inhibits plasma cell differentiation and has the potential to be used to treat SLE (33). A BRD4 inhibitor (Mivebresib) alleviates SLE-associated diffuse alveolar hemorrhage by interrupting the p300/BRD4/HIF1A axis thereby inhibiting M1 polarization (34). Here, we report that miR-146a inhibits expression of BRD4. Thus, BRD4 overexpression counteracted the effect of BG on LPS-induced THP-1 macrophage polarization and autophagy, and increased the level of inflammatory factors reduced by BG.

The findings of this study indicate that the BG suppresses M1 polarization and promotes M2 polarization of infiltrating macrophages in lupus-prone mice, thereby ameliorating disease symptoms, at least in part through the upregulation of miR-146a and subsequent targeting of BRD4. While our *in vitro* loss-of-function and rescue experiments support a functional link within this BG–miR-146a–BRD4 axis, it must be explicitly noted that certain aspects of the mechanism remain to be fully delineated. Specifically, the precise molecular interactions by which BG upregulates miR-146a, the direct functional consequences of BRD4 inhibition in this context, and the potential upstream signals that might modulate this pathway *in vivo* are not yet completely elucidated. These represent important avenues for future investigation and are acknowledged as a limitation of the current work.

## 5 Conclusion

In summary, we validated the therapeutic efficacy of BG in SLE mice *in vivo* and analyzed its effects on macrophages *in vitro*, elucidating the underlying mechanisms. BG suppresses M1 polarization and promotes M2 polarization of infiltrating macrophages in the kidney and spleen of lupus-prone mice by upregulating miR-146a, which targets BRD4. Furthermore, BG activates macrophage autophagy through the miR-146a/BRD4 axis, thereby inhibiting the secretion of inflammatory cytokines and production of autoantibodies. The anti-inflammatory and immunomodulatory effects of BG highlight its potential as a novel therapeutic agent for SLE.

## Data Availability

The datasets presented in this study can be found in online repositories. The names of the repository/repositories and accession number(s) can be found in the article/**Supplementary Material**.

## Supplementary material

Supplementary Figure 1 **(A)** WB used to detect the expression of autophagy-related proteins in kidney tissues. **(B)** WB used to detect the expression of autophagy-related proteins in spleen tissues.

Supplementary Figure 2 WB was used to detect the expression of autophagy-related proteins LC3 B and SQSTM1. **P* < 0.05, ***P* < 0.01.

Supplementary Figure 3 The expression of autophagy-related protein LC3B was detected by immunofluorescence. **P* < 0.05.

Supplementary Figure 4 The expression of autophagy-related protein SQSTM1 was detected by immunofluorescence. **P* < 0.05.

Supplementary Figure 5 The number of autophagosomes in each group was detected by transmission electron microscopy.

Supplementary Figure 6 The expression of miR-146a after cell transfection. **P* < 0.05.

Supplementary Figure 7 Immunofluorescence was used to detect the expression of LC3B after cell transfection. **P* < 0.05, **P < 0.01.

Supplementary Figure 8 Immunofluorescence was used to detect the expression of SQSTM1 after cell transfection. **P* < 0.05, ***P* < 0.01.

Supplementary Figure 9 The expression of miR-146a after cell transfection. **P* < 0.05.

Supplementary Figure 10 Immunofluorescence was used to detect the expression of LC3B after cell transfection. **P* < 0.05, ***P* < 0.01.

Supplementary Figure 11 Immunofluorescence was used to detect the expression of SQSTM1 after cell transfection. **P* < 0.05, ***P* < 0.01, ****P* < 0.001.

Supplementary Figure 12 Immunofluorescence was used to detect the expression of BRD4 after cell transfection. **P* < 0.05, ***P* < 0.01, ****P* < 0.001.

Supplementary Figure 13 The expression of LC3B and SQSTM1 after transfection was detected by WB. **P* < 0.05, ***P* < 0.01, ****P* < 0.001.
