# Supplementary material for: Bitter gourd peptides (BG) alleviate lupus progression in mice through regulation of miR-146a/BRD4 axis in macrophages
Source: Front Immunol. 2026 Feb 13;17:1666212. doi: 10.3389/fimmu.2026.1666212 (PMC12946005; doi:10.3389/fimmu.2026.1666212)
Supplement: Supplementary Figure 1 — (A) WB used to detect the expression of autophagy-related proteins in kidney tissues. (B) WB used to detect the expression of autophagy-related proteins in spleen tissues. [file Table1.doc]

**Bitter gourd peptides (BG) alleviate lupus progression in mice through regulation of miR-146a/BRD4 axis in macrophages**

Yu Wu ^&^, Wenyan Han^1^. ^&^, Xian Li^*^, Xiulan Su^*^

Clinical Medical Research Center of the Affiliated Hospital, Inner Mongolia Medical University, 010050 Hohhot, China.

^1^Clinical Laboratory, the Second Affiliated Hospital of Inner Mongolia Medical University, 010050 Hohhot, China

& Contributed equally first author

*Co-corresponding authors

*Correspondence should be addressed to Xian Li: [li_xian1214@163.com;](mailto:li_xian1214@163.com;) and Xiulan Su: [xlsu11@nmgfy.com](mailto:xlsu11@nmgfy.com)

**Supplemental Figure:**


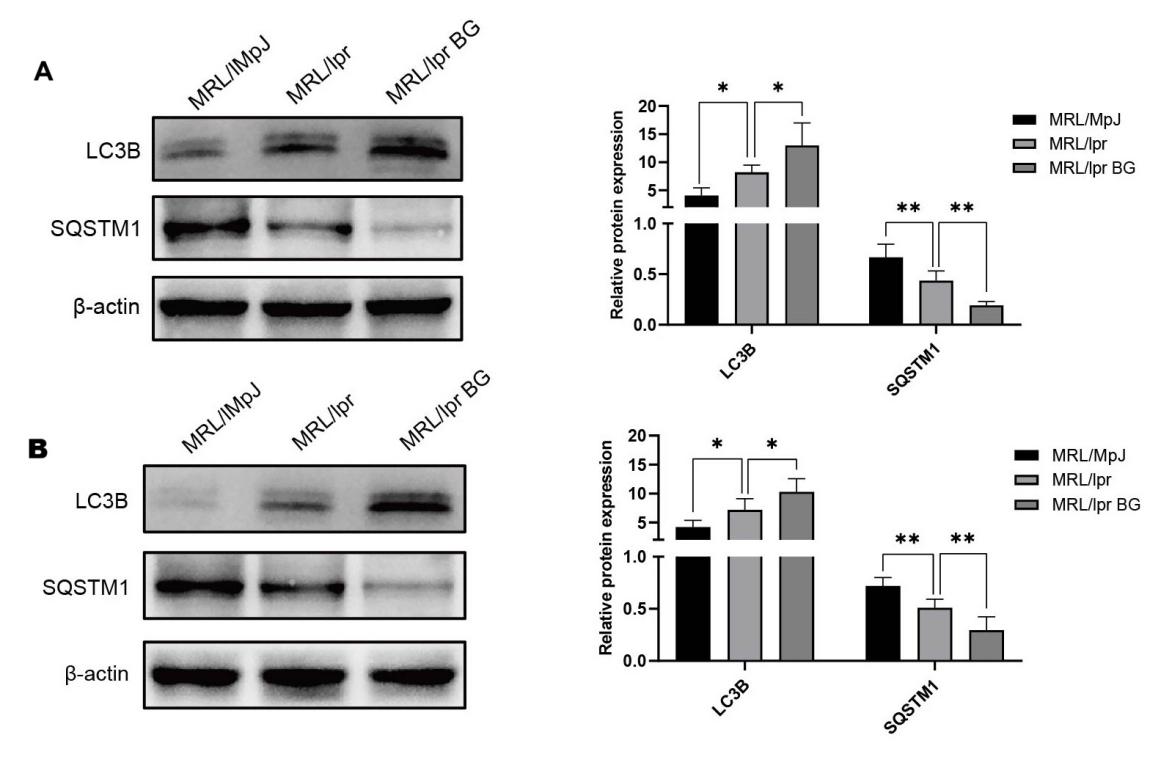


**Supplemental Fig. 1.** (A) WB used to detect the expression of autophagy-related proteins in kidney tissues. (B) WB used to detect the expression of autophagy-related proteins in spleen tissues.


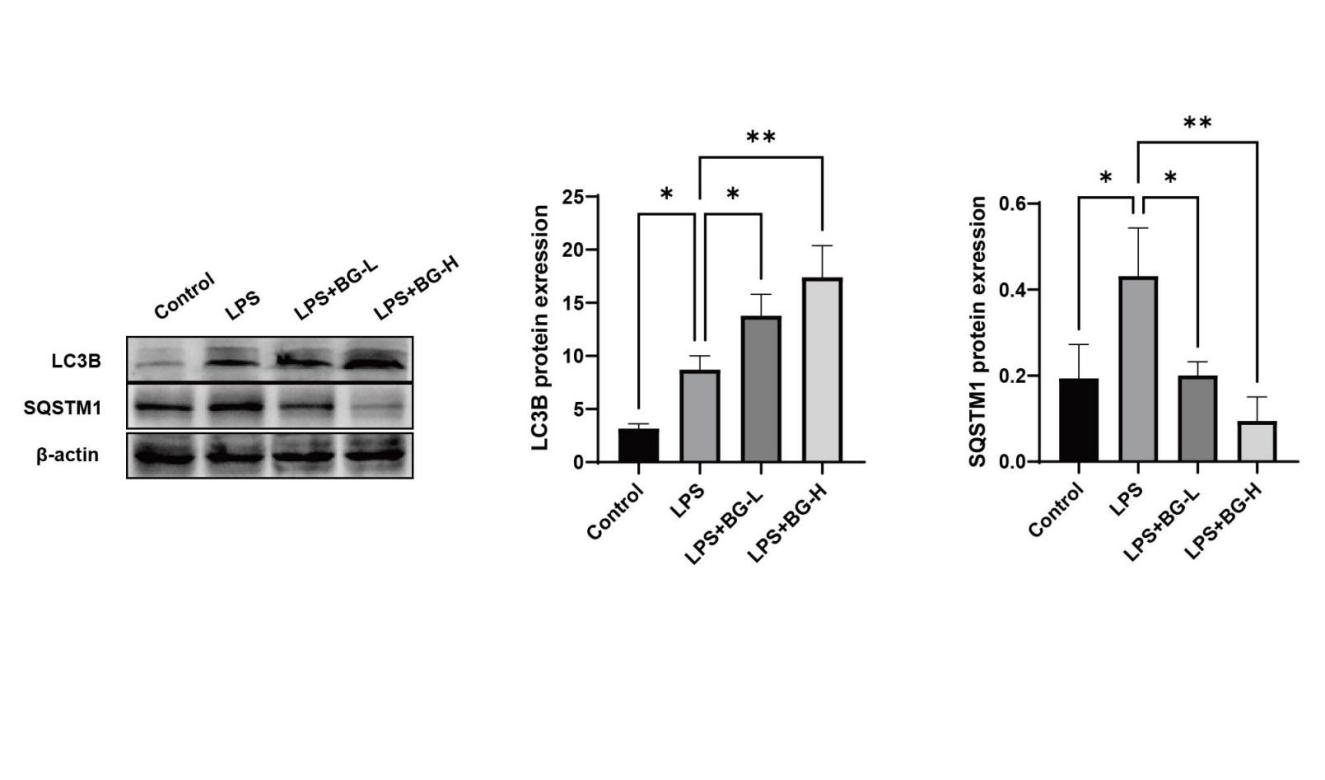
**Supplemental Fig. 2.** WB was used to detect the expression of autophagy-related proteins LC3 B and SQSTM1. **P* < 0.05, ***P* < 0.01.


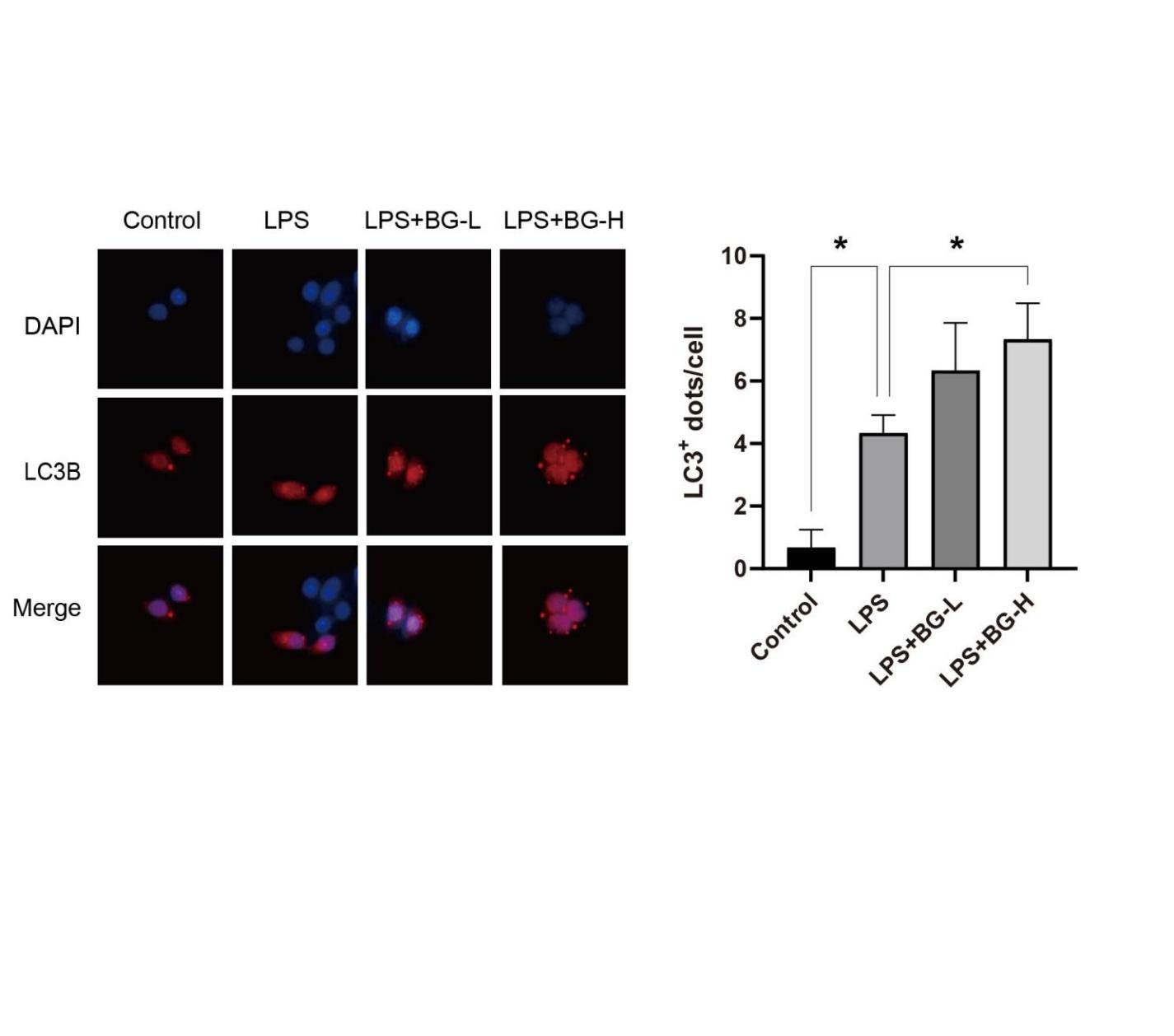


**Supplemental Fig. 3** The expression of autophagy-related protein LC3B was detected by immunofluorescence. **P* < 0.05.


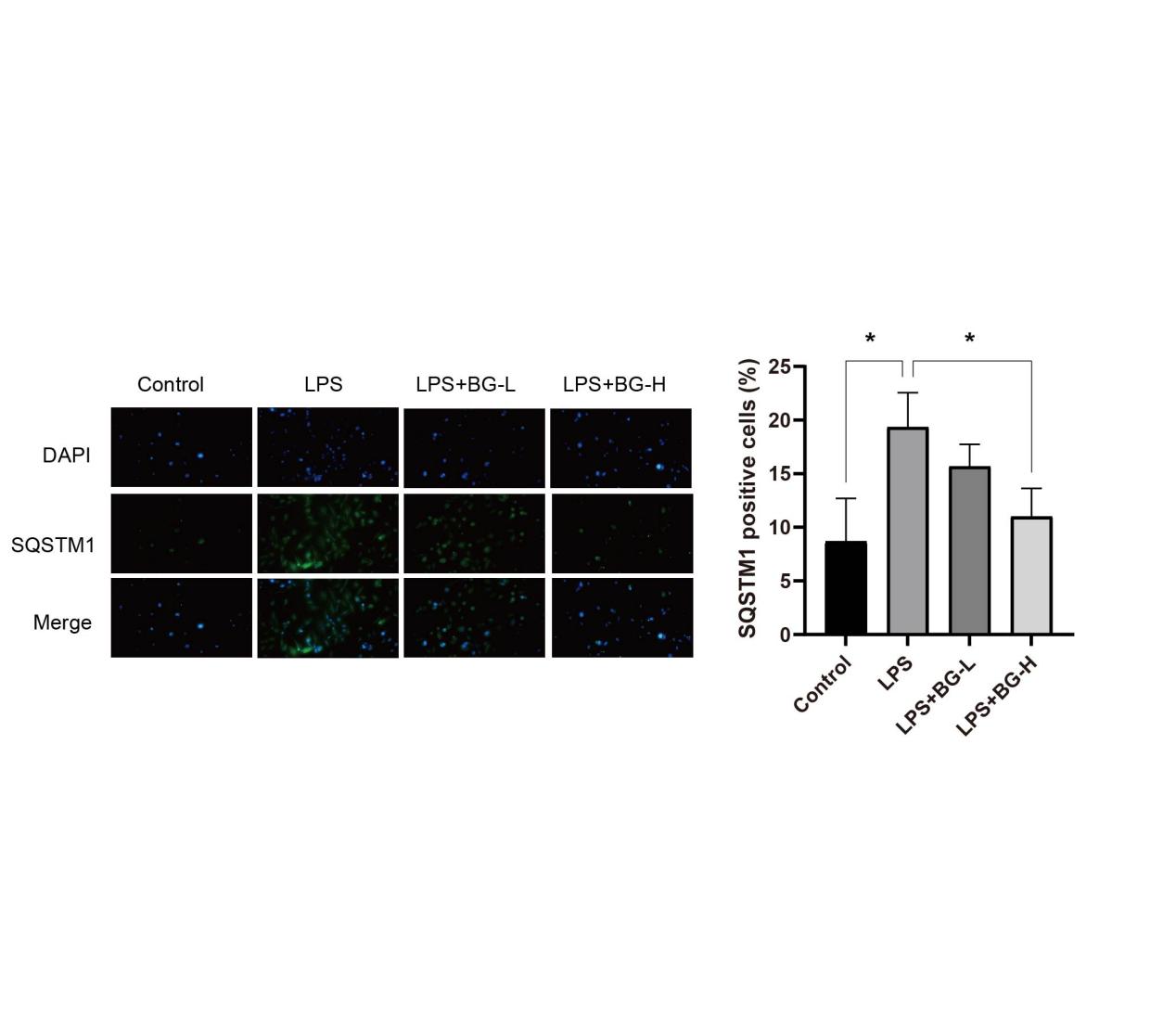


**Supplemental Fig. 4** The expression of autophagy-related protein SQSTM1 was detected by immunofluorescence. **P* < 0.05.


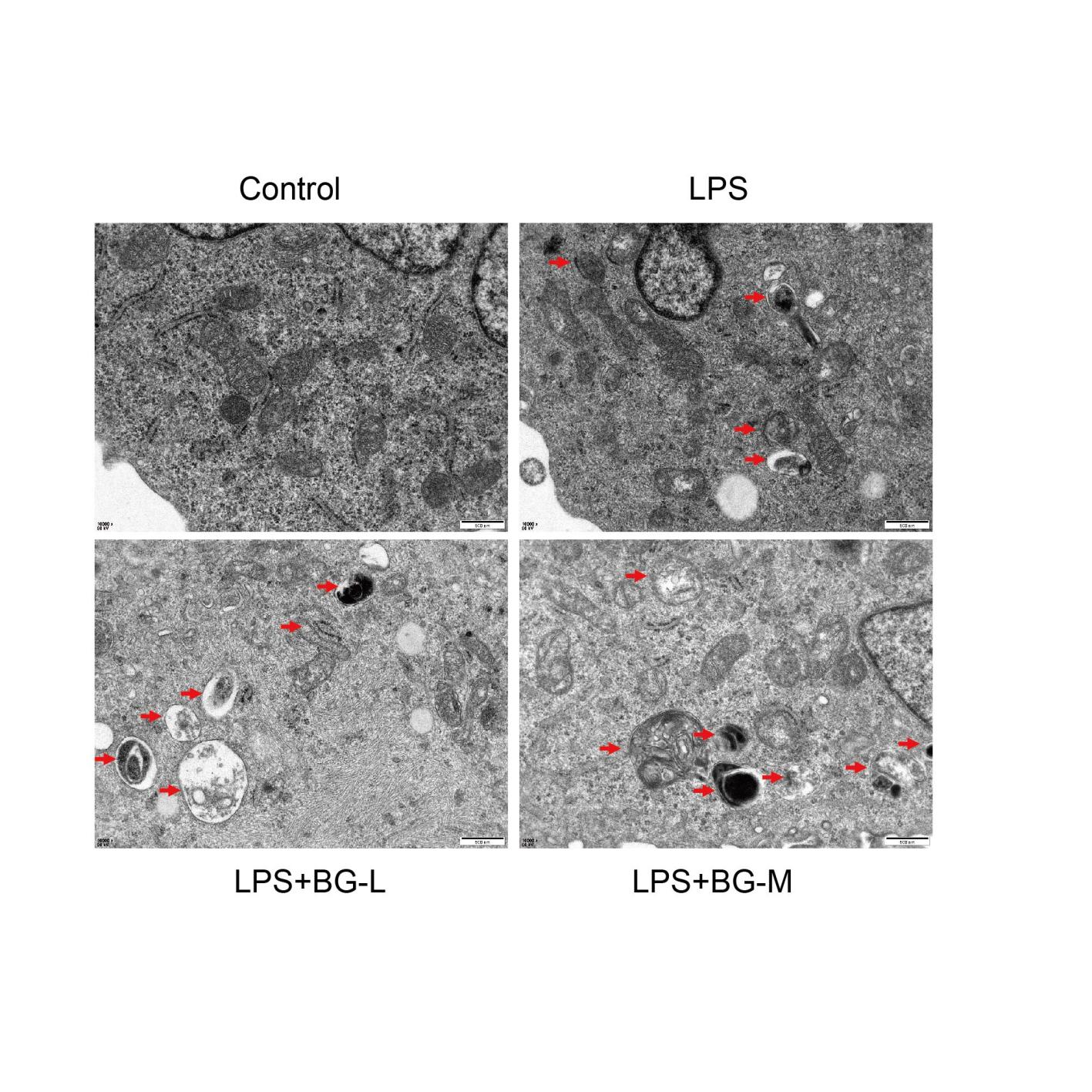


**Supplemental Fig. 5** The number of autophagosomes in each group was detected by transmission electron microscopy.


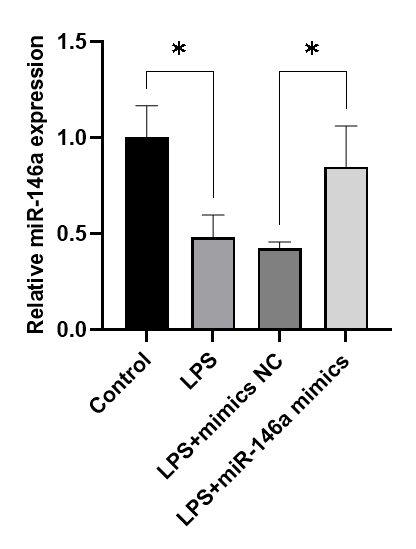


**Supplemental Fig. 6** The expression of miR-146a after cell transfection. **P* < 0.05.


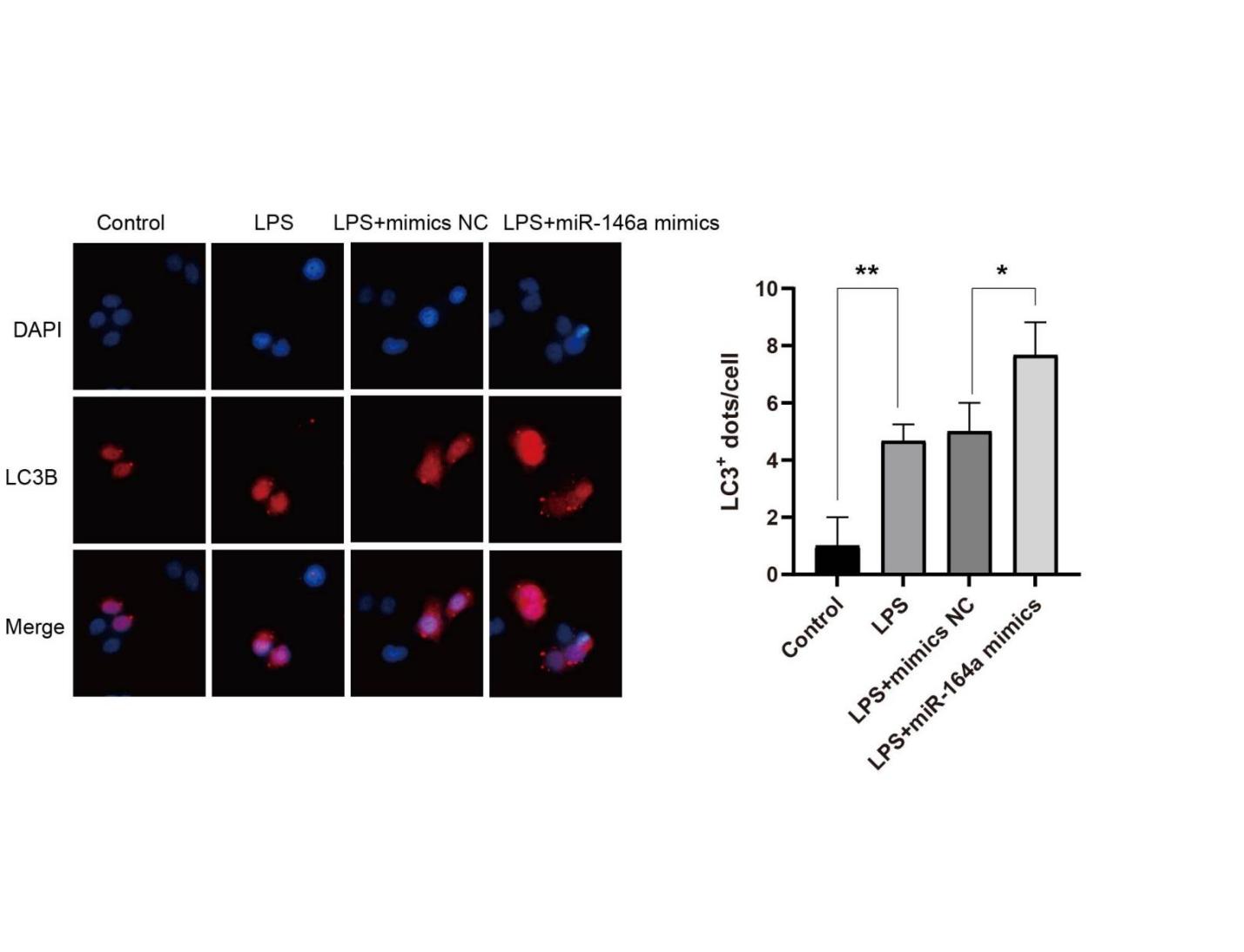


**Supplemental Fig. 7** Immunofluorescence was used to detect the expression of LC3B after cell transfection. **P* < 0.05, **P < 0.01.


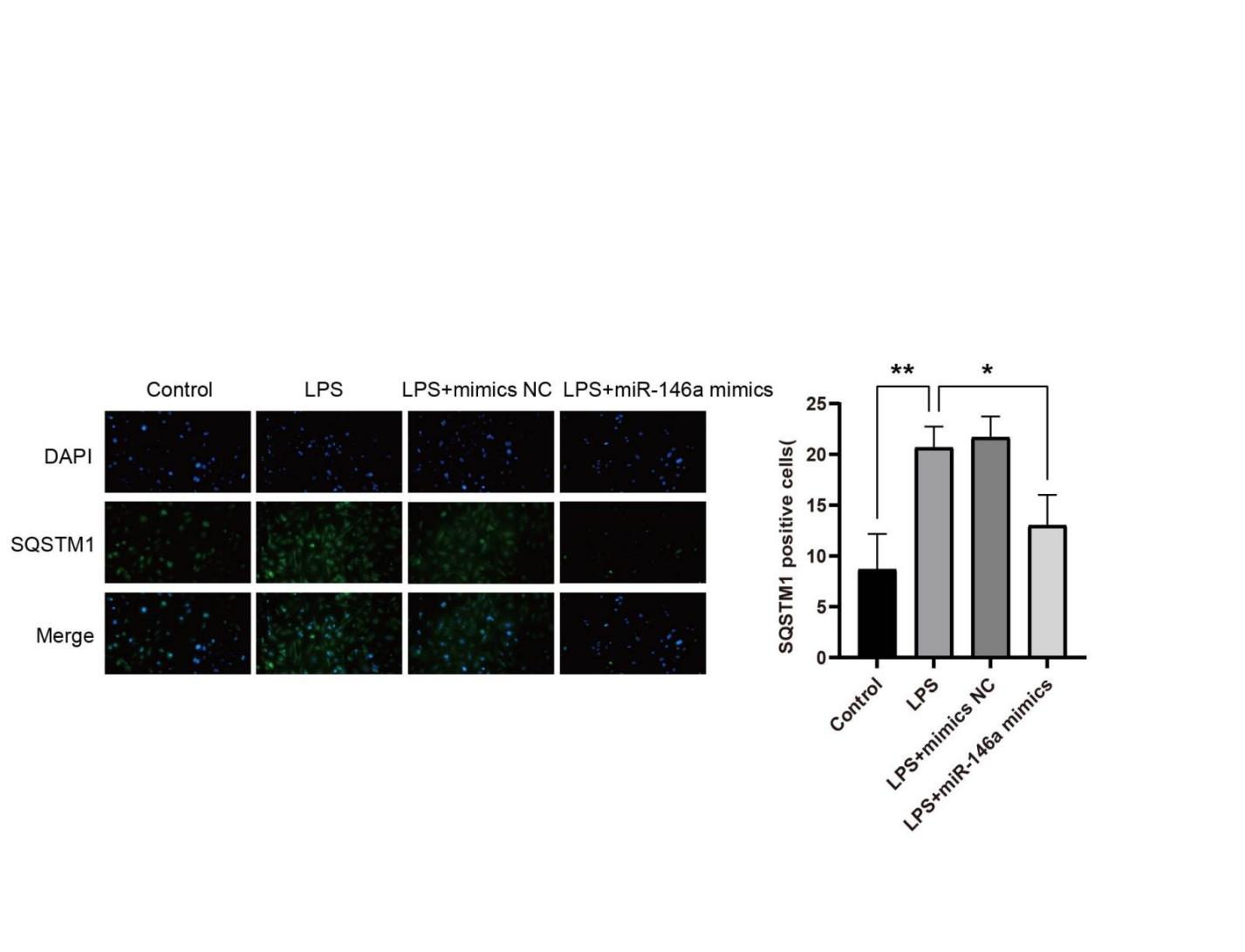


**Supplemental Fig. 8** Immunofluorescence was used to detect the expression of SQSTM1 after cell transfection. **P* < 0.05, ***P* < 0.01.


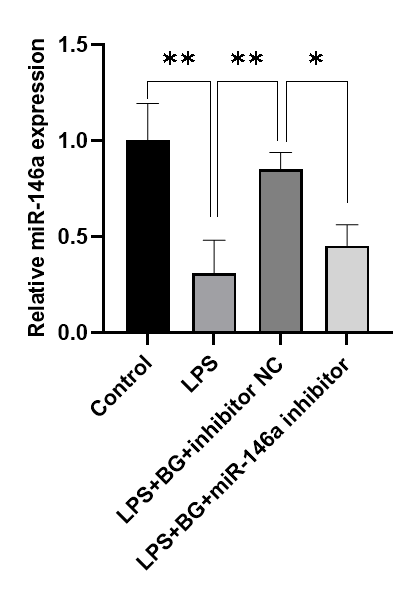


**Supplemental Fig. 9** The expression of miR-146a after cell transfection. **P* < 0.05.


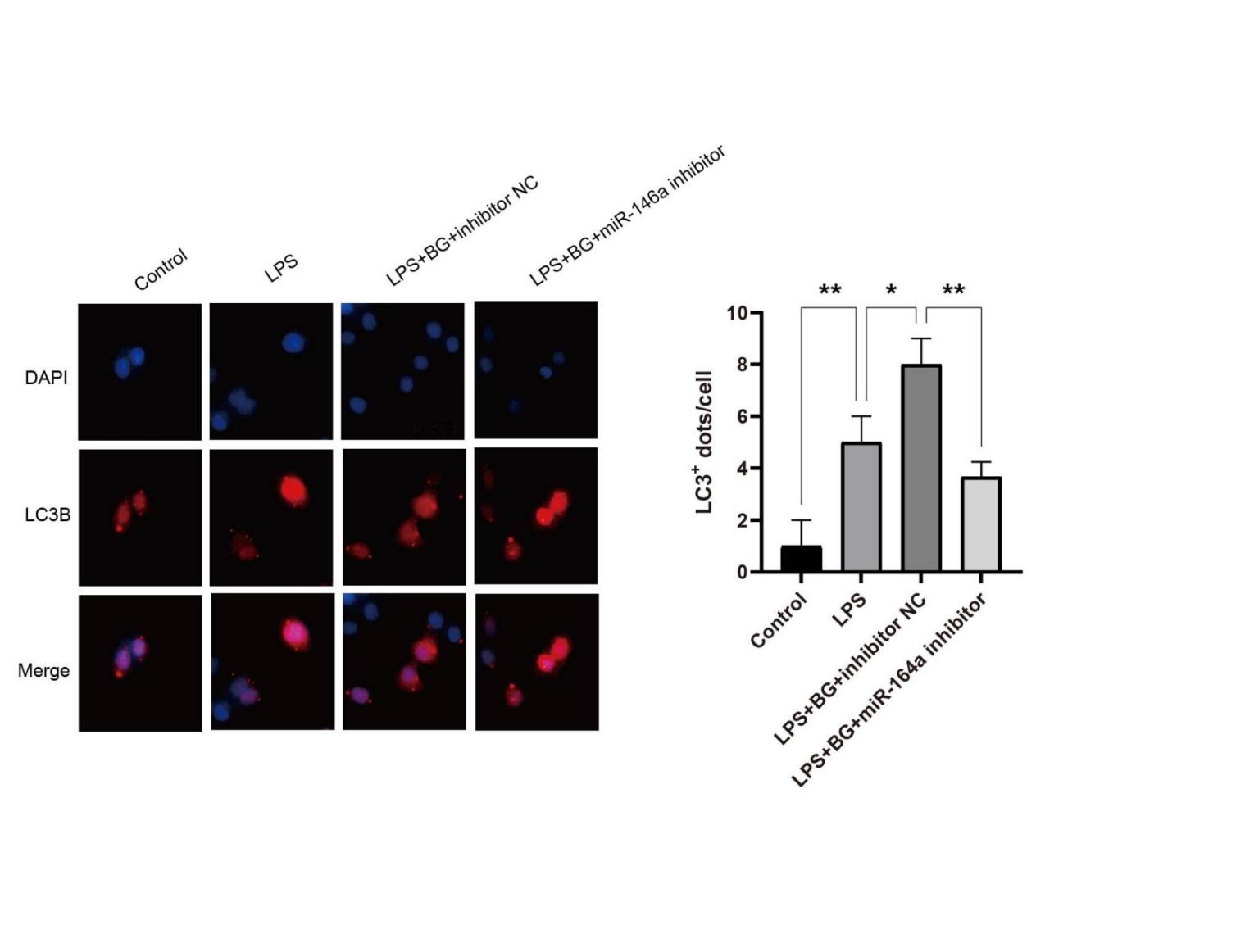


**Supplemental Fig. 10** Immunofluorescence was used to detect the expression of LC3B after cell transfection. **P* < 0.05, ***P* < 0.01.


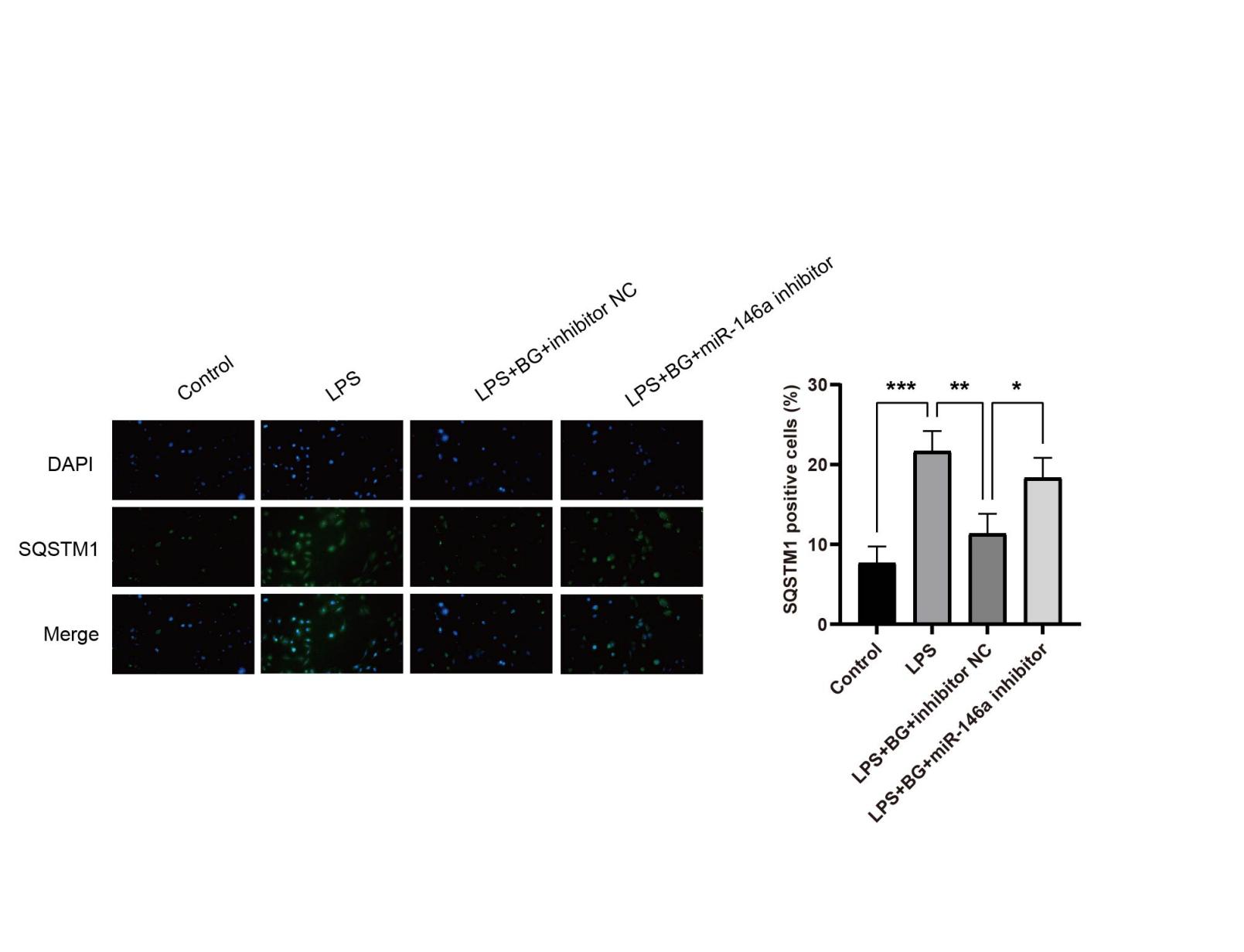


**Supplemental Fig. 11** Immunofluorescence was used to detect the expression of SQSTM1 after cell transfection. **P* < 0.05, ***P* < 0.01, ****P* < 0.001.


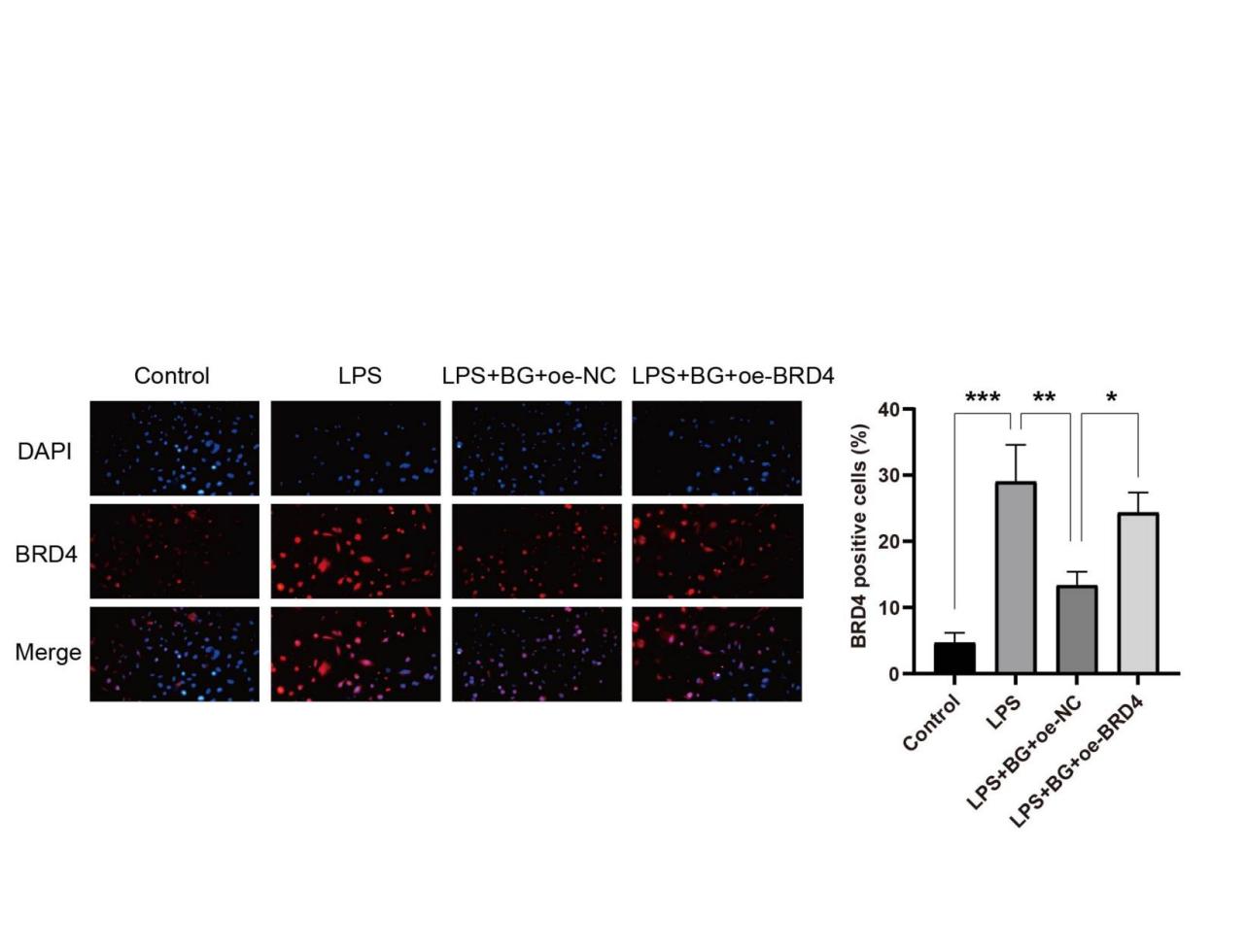


**Supplemental Fig. 12** Immunofluorescence was used to detect the expression of BRD4 after cell transfection. **P* < 0.05, ***P* < 0.01, ****P* < 0.001.


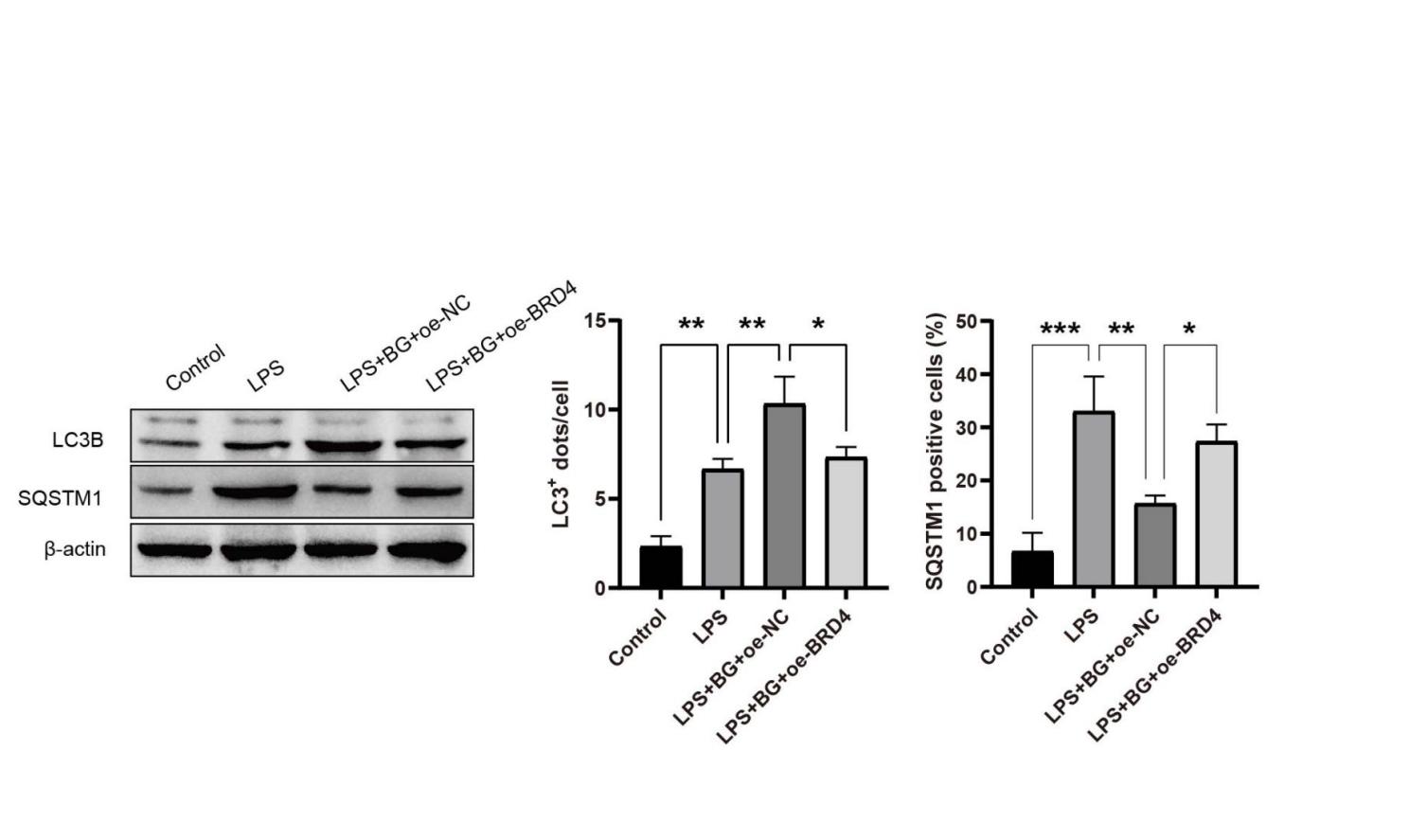


**Supplemental Fig. 13** The expression of LC3B and SQSTM1 after transfection was detected by WB. **P* < 0.05, ***P* < 0.01, ****P* < 0.001.
